# Continental‐Scale Evidence of Farm Management Impacts on Soil Carbon

**DOI:** 10.1111/gcb.70913

**Published:** 2026-05-15

**Authors:** Julian Helfenstein, Nick van Dijk, Anna Edlinger, Gabriel Y. K. Moinet, Sophie Q. van Rijssel, Alexandre M. J.‐C. Wadoux, Rachel Creamer, Carmen Vazquez, Vera L. Mulder

**Affiliations:** ^1^ Soil Geography and Landscape Group Wageningen University & Research Wageningen the Netherlands; ^2^ Wageningen Environmental Research Wageningen University & Research Wageningen the Netherlands; ^3^ Soil Biology Group Wageningen University & Research Wageningen the Netherlands; ^4^ College of Science and Engineering James Cook University Cairns Queensland Australia

**Keywords:** agricultural management, carbon farming, climate‐smart farming, soil carbon dynamics, soil organic carbon stocks

## Abstract

There are high expectations that agricultural practices can mitigate climate change and improve soil health by increasing soil organic carbon (SOC) stocks. However, existing large scale SOC monitoring treats agricultural management as a black box, meaning that observed patterns and trends cannot inform on the option space of agricultural practices to improve or deteriorate SOC stocks. Here, we combine for the first time management data from large scale systematic farm surveys (*n* = 248,362 farms) and representative soil monitoring data (*n* = 8834 locations) to quantify the impact of agricultural practices on three SOC metrics across all pedoclimatic zones of Europe (EU + UK): stocks, stocks relative to pedoclimatic benchmarks, and yearly change in SOC concentration. Our findings show that in arable and tree crops, but not in grasslands, management intensity is a significant contributor to SOC loss, with impact varying by soil and climate region. However, we also observed that several practices (e.g., high share of manure, organic management, and a high proportion of leys in crop rotation) demonstrated potential for increasing SOC stocks. Under a scenario where all agricultural land in Europe would be managed as that of the 10% most optimally managed farms in terms of SOC benefit, SOC stocks would increase by 1.58 Pg C across Europe (95% CI: 1.27–1.89 Pg C). Whereas under a scenario where farms are managed as the 10% least optimally managed farms, SOC would decrease by −0.92 Pg C (−1.15 to −0.68 Pg C). However, it is important to note that these estimates reflect steady‐state SOC stocks only (i.e., they do not represent the transient build‐up or loss over time, or interactions with a changing climate). This paper thus quantifies how agricultural practices influence patterns in SOC stocks at the continental scale, identifying leverage points for site‐specific policies to improve SOC stocks.

## Graphical abstract

Using standardized European farm surveys (*n* = 248,362 farms) to predict farm management at every soil monitoring location (*n* = 8834) allowed quantifying impacts of agricultural practices on SOC stocks, and changes thereof. Taken together, the option space for agricultural practices to impact SOC stocks in EU + UK ranged from −0.92 to 1.58 Pg C, with management effects varying for croplands, grasslands, and tree crops by pedoclimatic zone.

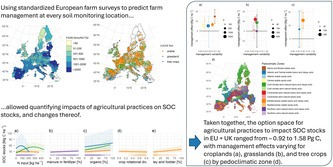

## 1 Introduction

Maintaining soil organic carbon (SOC) stocks in agricultural systems is critical for sustaining soil multifunctionality and avoiding CO_2_ emissions from degradation (Beillouin et al. 2023; Roe et al. 2019; Smith 2008). Protecting existing SOC helps preserve nutrient cycling, soil structure, biodiversity, and water regulation, all of which underpin productive and sustainable farming systems (van Rijssel et al. 2025). Increasing SOC stocks, defined as soil carbon sequestration (Don et al. 2024), can also remove CO_2_ from the atmosphere, but global sequestration potential is modest and time‐limited due to steady states, reversibility, and socio‐economic constraints (Moinet et al. 2023). Even optimistic scenarios suggest that it could offset only a small share of the emission reductions needed for climate targets (Roe et al. 2019), and this potential reduces further when deployment of SOC sequestering practices is adjusted to minimize trade‐offs with production (McClelland et al. 2025). Nonetheless, optimal SOC management is a central component of sustainable soil management and sits on the right side of the climate equation. A significant amount of research has shown that the adoption of sustainable management practices such as diverse crop rotations, organic amendments, or perennial cropping—among others—could slow or reverse declines in SOC stocks (Beillouin et al. 2023; Wiesmeier et al. 2019). Large gaps remain, however, in our understanding of the effect of practices on SOC dynamics in commercial farms, which combine broad sets of these practices in complex management systems.

Indeed, current understanding of management effects on SOC stocks mostly stems from controlled field experiments (López i Losada et al. 2025; Tang et al. 2025; Wiesmeier et al. 2019), which reveal that both management effects on SOC and the influence of SOC on other ecosystem functions are highly context dependent (Moinet et al. 2023; Schepens et al. 2025). Meta‐analyses of dozens of controlled experiments allow us to isolate the influence of pedoclimatic variability and have shown that biochar applications, organic fertilizers, perennial crops, and agroforestry have positive effects on SOC in a large majority of cases, while tillage intensity, crop rotation, and mineral fertilizer application have smaller or more ambivalent effects (Bai et al. 2018; Beillouin et al. 2023; Poeplau and Don 2015). While these provide important insights, meta‐analyses of controlled experiments do not capture the complex interactions and variability of farming practices as observed in actual farms. This is because controlled experiments differ from on‐farm reality, as single practices are emphasized for testing, ignoring that these practices often come in bundles (Debernardini et al. 2025) and the distribution of controlled field experiments is haphazard rather than representative. A recent synthesis of management practices (tillage, crop rotation, and fertilization) identified more than 285,000 possible management bundles (Rillig and Lehmann 2019), the application of these bundles is then further multiplied by the differing pedo‐climatic conditions when considering the outcomes in terms of SOC stocks and stock change (Edlinger et al. 2023).

Opportunities for large‐scale assessments of SOC stocks have arisen through the increase in national and continental soil monitoring (Orgiazzi et al. 2018; Pan et al. 2010; Román Dobarco et al. 2023); however, to date these have not considered agricultural management. For example, analysis of changes in SOC stocks between 2009 and 2018 from the European soil monitoring network LUCAS Soil showed small increases in SOC stocks, especially in grassland soils (De Rosa et al. 2024). However, in this and other large‐scale SOC assessments (Levy et al. 2024; Wadoux et al. 2023; Xie et al. 2007), agricultural management was not considered. Therefore, while previous work monitors the change in SOC stocks, it cannot explain why patterns are observed, or specifically how agricultural practices influence the outcome. Clear understanding of how management impacts SOC stocks, and how that varies with pedoclimatic context, is essential for science‐informed policy, for example, defining sustainable management practices (Alblas et al. 2025). Thus there is an urgent need to integrate farm management and soil monitoring data to provide empirical evidence on the impact of real‐world management practices on SOC stocks.

Assessments of management impacts on SOC under actual farm conditions remain rare, primarily due to two major challenges: limited availability of farm management data and the high complexity of covarying factors (pedo‐climatic conditions, social influence, economic lock‐ins, etc.). The first challenge can be addressed by combining on‐farm soil sampling with farmer interviews to document management practices (Edlinger et al. 2025; Garland et al. 2021; Vahter et al. 2022). The second challenge can be mitigated either by limiting geographic scope to constrain variability, sometimes including a paired‐site approach where controls are adjacent to the studied practice (Moinet et al. 2025; Mudge et al. 2017; van Rijssel et al. 2025; Williams et al. 2020), or by focusing on specific cropping systems across broad pedoclimatic gradients (Edlinger et al. 2023). However, both of these challenges demand labor‐intensive approaches which restrict sample size and representativeness of earlier studies.

**FIGURE 1:**
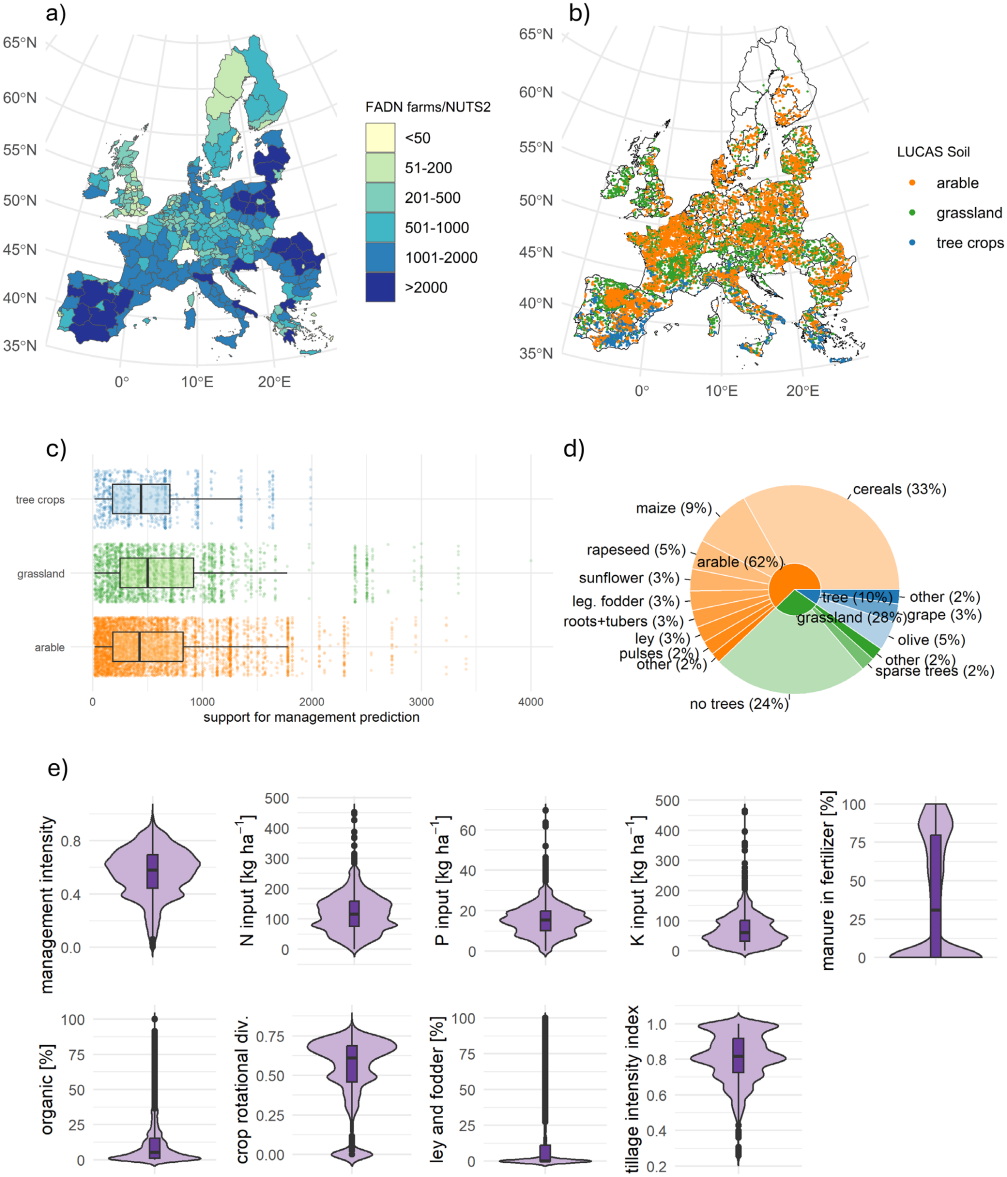
Combination of pan‐European farm and soil monitoring data. (a) individual farm data from the Farm Accountancy Data Network (FADN, *n* = 248,362 for the years 2018–2020), a representative farm survey conducted by the European Commission, was used to predict farm management at every sample location (b) of the European soil monitoring network (LUCAS Soil, *n* = 8834). (c) Management at every location was predicted using all farms growing the same crop and in the same NUTS2 region and altitude zone—see methods for deviations. (d) numerical proportion (%) of agricultural land cover types at LUCAS locations. (e) Distributions of key management variables as predicted at all LUCAS locations.

Here, we combined standardized European farm surveys (Farm Accountancy Data Network (FADN), *n* = 248,362) with large scale soil monitoring data to disentangle the effect of farm management practices on SOC across all 27 European Union member states plus the United Kingdom (Figure 1). While several studies have demonstrated the potential of FADN data to inform on spatial and temporal patterns in agricultural practices relevant for environmental performance on a case‐study basis; for example, in England (Westbury et al. 2011), Italy (Cardillo et al. 2023), or Lithuania (Dabkiene et al. 2021), analysis of agricultural practices using FADN data has not been done for the whole of Europe, nor has it been combined with soil data. We first used farm data to predict likely management practices at all agricultural sampling locations of the LUCAS Soil monitoring program (*n* = 8834) (European Commission 2025a). We then analyzed relationships between management and three SOC metrics: (1) SOC stocks, (2) deviations from benchmarked SOC stocks, and (3) the change in SOC concentration from 2009 to 2018—while taking variability in soil typology and climate into account. We hypothesized that:

1. Permanent crops, grasslands, and extensive crops (e.g., temporary grasslands, forage crops) are associated with more positive SOC outcomes than intensive crops (e.g., potatoes, sugar beet or cereals).
2. Higher management intensity negatively correlates with SOC outcomes.
3. The share of manure in the fertilizer regime, crop rotational diversity, share of leys in the crop rotation, and organic cultivation are positively associated with SOC outcomes, while tillage intensity is negatively associated.
4. Pedo‐climatic conditions significantly influence the relationship between management and SOC stocks.

In addition, we discuss what our findings on management effects on SOC stocks imply for the contribution of agricultural soils to the European C budget, by upscaling our results under two scenarios: (1) all fields managed like the current top 10% best managed fields and (2) all fields managed like the current bottom 10%.

## 2 Materials and Methods

### 2.1 Farm Management Data Source and Processing

Individual farm management data was obtained for the years 2018–2020 from the Farm Accountancy Data Network (FADN) through data access contract IFD 2023_06 with the Directorate‐General for Agriculture and Rural Development of the European Commission. FADN annually samples roughly 80,000 farms, representing a population of about 5 million farms in the EU. The survey is designed to be representative in terms of regions, economic farm sizes, and types of farming (European Commission 2025a). Type of farming refers to 14 classes ranging from ‘specialists cereals, oilseed, and protein crops’ to ‘specialist olives’ to ‘mixed livestock’ (European Commission 2025b). Because we used three survey years (2018–2020), our dataset comprised 248,362 farm‐year observations, and since farms cannot be tracked across years for privacy reasons, we simply referred to each observation as a farm in the rest of this text (Figure 1a). This assumption should not affect our analysis, since all predictions are based on at least 15 farms.

We calculated the following indicators for each farm:

- N, P, K nutrient input in kg ha^−1^ as the sum of mineral fertilizer plus animal‐based fertilizer. The latter was calculated as livestock density [livestock units ha^−1^] times the nutrient excretion per livestock unit per year. We assumed values for dairy cows equating to 135 kg N, 19 kg P, and 139 kg K dairy cow^−1^ year^−1^ because one dairy cow equals one standard livestock unit (Statistics Netherlands 2012). However, it is important to consider that nutrient excretion values depend on livestock type and production system (Statistics Netherlands 2012).
- Share of manure in the fertilizer mix as animal‐based fertilizer divided by total nutrient input.
- Probability of organic farming, which was calculated as the area under organic cultivation divided by the total area for each crop, region, and altitude class combination. The analysis did not distinguish between under‐conversion and certified organic farms.
- Crop rotational diversity as the Gini‐Simpson index of arable crop areas per farm (Helfenstein et al. 2022).
- Crop rotational composition as the share of each crop category, that is, cereals, roots and tubers, dry pulses, sunflower, rapeseed, other industrial crops, fodder legumes, maize, vegetables, and leys. The crop rotational diversity was calculated following Ballot et al. (Ballot et al. 2023), with some minor modifications. First, while Ballot et al. (2023) only included the most important crops, we included all reported crops. Second, we created a separate crop category for fodder legumes.
- Tillage intensity was calculated as fraction of area under conventional tillage + fraction of area under conservation tillage * 0.2 + fraction of area under zero tillage × 0 (Holland 2004). Since FADN does not contain data on tillage, tillage data was acquired from EUROSTATS for 2016 (EUROSTAT 2020). The data contain the areas of conventional, conservation, and zero tillage for each NUTS2 region, farm size category, and farm type combination, allowing for the calculation of 8087 unique values of tillage intensity. This data was combined with the FADN dataset based on NUTS2 region, farm size, and farm type to predict farm specific tillage intensity.

### 2.2 Soil Organic Carbon Data Source and Processing

Soil data was derived from Land Use/Cover Area frame statistical Survey Soil (LUCAS Soil), the European soil monitoring survey, for the years 2009/2012 and 2018 (Figure 1b). LUCAS Soil is a standardized and systematic topsoil survey across the European Union and United Kingdom with roughly 22,000 sample locations, around half of which on agricultural land (Orgiazzi et al. 2018). In addition to land use (arable, tree crop, or grassland), the exact land cover, that is, crop type is recorded at every sample location (Figure 1d). We calculated three SOC metrics to provide an overview of the impact of management on SOC dynamics: SOC stocks, benchmarked SOC stocks, and the change in SOC concentration from 2009/2012 to 2018.

SOC stocks were calculated using SOC concentration and bulk density data from 2018. A true equivalent soil mass (ESM) approach was not possible in the absence of depth‐layered data on SOC concentration and bulk density (Fowler et al. 2023). To allow for unbiased comparisons of SOC stocks across land uses that differ in bulk density, we adopted a mass‐standardized fixed‐depth approach: we predicted a reference fine soil bulk density (B_d, ref_) from edaphic covariates and used it to compute 0–20 cm SOC stocks for all points, including those lacking measured bulk density (B_d_).

Measured fine soil B_d_ (< 2 mm) was available for about a third (*n* = 3538) of the sampling points across arable, grassland and permanent crops. Using this subset, we first developed land‐use–specific regression models predicting B_d_ from a subset of the soil variables from the LUCAS Soil database (van Leeuwen et al. 2024). To avoid multicollinearity, we selected edaphic properties that were uncorrelated between them, including soil texture (percentage sand and clay), total nitrogen, pH, and mean annual precipitations (MAP). As the purpose of predicting B_d_ was to calculate SOC stocks, we excluded OC from all models. This methodological choice likely reduced our predictive power but avoided circularity: including OC as a predictor of B_d_ and then multiplying predicted B_d_ by OC to calculate SOC stocks (Equation 1) means OC appears twice in the stock calculation, potentially biasing land‐use comparisons. We then evaluated all candidate combinations and compared models using AIC. The parameter estimates were extracted from the best regression model (including pH, total N and the interaction between sand and clay) and were used to calculate land‐use–adjusted reference bulk density (B_d, ref_) for each sampling point of the whole dataset (*n* = 10,523). The reference bulk density was then used to calculate SOC stocks, accounting for the volume proportion of coarse fragments (C_f_) following (Poeplau et al. 2017), as:

(1) $$\begin{array}{c} \mathrm{SOC}\;\text{stocks}\;\left(\mathrm{Mg}\;C\;{\mathrm{ha}}^{-1}\right)=B_{d,\mathrm{ref}}\;\left(g\;{\mathrm{cm}}^{-3}\right)\\ \;\times \mathrm{OC}\;\left(\mathrm{mg}\;C\;g^{-1}\right)\times 20\;\left(\mathrm{cm}\right)\times 0.1\times \left(1-C_{f}\right) \end{array}$$

Comparison between measured B_d_ and predicted B_d, ref_ can be found in Figure S1 and was comparable to (De Rosa et al. 2024). Benchmarked SOC stocks were calculated as the observed SOC stocks divided by the pedoclimatic average, or typical, values from Feeney et al. (Feeney et al. 2024). Since our dataset was larger than the one used in earlier work, 19% of sample points did not have an assigned pedoclimatic zone. For these missing values, the pedoclimatic zone was predicted using a random forest classification model. The model was fit using the ranger package (Wright and Ziegler 2017) on the training data (*n* = 8587), for which pedoclimatic zone was known. The model had 500 trees, a minimum node size of 1, and a mtry parameter of 3, and the following predictor variables were used: SOC concentration, NUTS3 administrative region, land cover, lithology, sand, silt, clay, aridity index, potential evapotranspiration, Köppen‐Geiger climate class, mean annual temperature, and mean annual precipitation. See Table S4 for data sources. The out‐of‐bag error rate was 0.057.

The change in SOC concentration was calculated as the difference in SOC concentration for 2018 and 2009/2012, divided by the number of years (De Rosa et al. 2024).

Data cleaning followed De Rosa et al. (2024). Firstly, organic soils with > 160 g C kg^−1^ were removed. Since land use change (e.g., from grassland to cropland) has been reported to strongly affect SOC dynamics (De Rosa et al. 2024), locations which were not consistently the same land cover class (grassland, permanent crop, or arable) were removed. Finally, change in SOC values that were > 100% relative change from 2009 to 2018 or > 3 g kg^−1^ year^−1^ absolute change were removed, because such large changes imply either measurement uncertainty or soil movement (De Rosa et al. 2024). This decreased sample size to 8706 for concentration of SOC in 2018, 6795 for (benchmarked) SOC stocks, and 6362 for change in SOC concentration, respectively.

### 2.3 Predicting Farm Management at Every Soil Sample Location

Since the exact location of each farm is not known, predicting farm management at every soil sampling location was done based on common NUTS2 region, altitude class (< 300 m, 300–600 m, > 600 m), and crop type. Farm management data was linked to LUCAS Soil sample points in one of three ways, depending on data availability. For sample locations where there were more than 15 farms cultivating the given crop in the same NUTS2 region and altitude class in the FADN dataset, those farms were used to predict the management indicators using Equation (2). This approach could be applied to 73.6% of LUCAS sample points. For the remaining points, the search radius for farms was extended by ignoring altitude within the same NUTS2 region. In this way, management could be predicted at an additional 22.9% of points. If there was still no match, the geographically closest farms with the same land cover class within the same country were used (an additional 2.0%). A remaining 1.5% (*n* = 105) of LUCAS points could not be matched with FADN data after all three steps, for which management was set as NA. Since this approach allowed disentangling specific management regimes for 36 different crop and grassland types within each region and altitude class based on extensive empirical data, it provides considerably more nuanced estimates of agricultural practices compared to earlier studies that relied on regional or national averages or did not differentiate management between crops.

(2) $$w_{i}=\text{sqrt}\left(\frac{\mathrm{are}a_{i}}{\mathrm{UA}A_{i}}\times \frac{\mathrm{are}a_{i}}{\text{total area}}\right)$$

This weighting scheme accounts both for a farm's specialization in a given crop (first term) and its representativeness in the regional production of that crop (second term). Hence, management indicators were aggregated for every NUTS2 region, altitude class and crop type combination, for which there were at least 15 farms in the dataset. Values for years 2018–2020 were used to account for annual variation. The median number of farms used to predict management for every soil sampling location was 426 for arable, 440 for tree crops and 500 for grasslands (Figure 1c). The cut‐off of 15 farms was prescribed by the European Commission to safeguard the anonymity of farms. Each management indicator was calculated using weighted medians and weighted standard deviations using the sjstats package in R (Lüdecke 2022). Weights were calculated considering both the proportion of the crop in the total farm area and the proportion of the farm's crop area relative to the total area of that crop in the region (Equation 2).

Where *w*_*i*_ is the weight of each farm. *area*_*i*_ is the area of the crop on the farm and *UAA*_*i*_ is the total utilized agricultural area of the farm. Total area is the sum of all crop areas for that crop ($\sum \mathrm{are}a_{i}$). Weighing by the proportion of crop in the farms UAA increases accuracy of predicting crop‐specific management, and weighing by the proportion of crop grown on the farm relative to the total area grown in the region accounts for the representativeness of the farm for the region. This way, each management indicator was predicted at every LUCAS soil sampling location (Figure 1e).

To assess the performance of the predictions, that is, to quantify if our approach provided more precise estimates of management than NUTS2 level averages, Kruskal‐Wallis rank sum test was used, a robust alternative to one‐way ANOVA (Hollander and Wolfe 1973). *χ* ^2^‐statistic and *p*‐value were calculated for each management indicator per NUTS2 region. In the main text we report the percentage of NUTS2 regions with a significant Kruskal‐Wallis rank sum test (*p* < 0.05) for each management indicator, that is, where our approach based on individual farm data successfully captures a significant portion of intra‐regional variability.

### 2.4 Management Effect on Soil Organic Carbon

Impact of agricultural practices on SOC was assessed using mixed linear models with pedoclimatic zones as a random effect and management indicators as fixed effects. Since SOC dynamics are known to differ by soil and climate type (Wiesmeier et al. 2019), mixed linear models were used because they allow testing hypotheses while controlling for variability associated with different pedoclimatic conditions, thereby improving the robustness and generalizability of the results.

Three separate linear mixed‐effects models were fitted for each SOC metric. The first model assessed the effect of overall management intensity, the second examined the influence of individual agricultural practices across all land‐use types, and the third focused specifically on arable crops, including practices such as crop rotation that are not applicable to grasslands or permanent crops. For each case, a full model incorporating all relevant management variables was initially specified. Model simplification was performed using stepwise backward elimination of non‐significant terms via the step() function from the *lmerTest* package (Kuznetsova et al. 2017). The significance of the final model was evaluated by comparing it to a random‐effects‐only model using likelihood ratio tests (ANOVA). Marginal effects were derived using the ggpredict() function from the *ggeffects* package (Lüdecke 2018).

SOC stocks were log +1 transformed, benchmarked SOC stocks were log‐transformed, and change in SOC was hyperbolic arc‐sine transformed to better meet assumptions of normality.

The management intensity index was calculated as the average normalized intensity rank of the following management indicators: probability of organic farming, crop richness, crop rotational diversity, share of manure in the fertilizer mix, nutrient input, tillage intensity, and share of ley and fodder legumes in the crop rotation (Helfenstein et al. 2022; van Rijssel et al. 2025). For each variable, farms were first ranked across the dataset using within‐variable ranks while retaining missing values. Variables representing practices expected to reduce intensity (organic, crop richness, crop rotational diversity, share of manure in the fertilizer mix, and share of ley and fodder legumes) were ranked in reverse order. Crop diversity (Gini Simpson and richness), nutrient input (N, P, K), and share of manure (N, P, K) were represented by multiple indicators in our data. In these cases, the measured indicator values were weighted so that each function received unit weighting on average, with equal contributions from all constituent indicators (van Rijssel et al. 2025). Each resulting ranked variable was rescaled to the 0–1 range using min–max normalization (based on non‐missing values). Finally, the intensity indicator was calculated for each farm as the unweighted mean of all normalized variables, ignoring missing values.

### 2.5 Soil Type‐Specific Management Effects

Since sensitivity of SOC to agricultural practices is known to differ based on soil, climate and land use (Moinet et al. 2023), we tested how management effects varied based on pedoclimatic zone. Management effect was defined as the difference in SOC stocks observed on the 10% most optimally managed and 10% least optimally managed sites per soil pedoclimatic zone. Optimal management was defined based on the expected SOC stocks according to the linear mixed models, that is, high probability of organic cultivation, high share of manure, and high share of leys in the rotation for arable; high probability of organic cultivation, high share of manure, and intermediate levels of N input for grassland and permanent crops (Figure 4 and Tables S1–S3). Note that optimal management was defined for expected impact on SOC stocks only, not considering agricultural production or other environmental outcomes.

To account for the fact that significant management effects were more likely to be observed in pedoclimatic zones with high variability in management, management effect was plotted as a function of management variability. Significant management effects for pedoclimatic zones with low management variability can be interpreted to denote particularly sensitive soils, since even small changes in management are likely to significantly affect SOC stocks.

### 2.6 Option Space for Agricultural Practices to Influence Soil Carbon Stocks

We defined the option space for agricultural practices to influence soil carbon stocks as the difference between two scenarios, one where all agricultural land within each pedoclimatic zone would be managed according to today's top 10% most optimally managed fields, and a second where all agricultural land is managed according to today's worst 10%. Best/worst management was based on the linear mixed models. In more detail, the final models were used to identify fields for which, based on their management, the model expects highest/lowest SOC stocks. The option space scenarios assume no change in land cover type (i.e., arable, grassland or tree crops), only changes in management. To calculate impacts of these scenarios on SOC stocks across Europe, pedoclimatic zone specific management effects were multiplied by their respective area (Table S5). Areas were calculated by intersecting land cover (arable, grassland, tree crops) and pedoclimatic zone maps. The land cover map was the CORINE Land Cover 2018 map, which we reclassified and extracted the arable cropland, permanent crops and grassland classes. Spatial data on pedoclimatic zones was taken from (Feeney et al. 2024). Uncertainty of scenarios was calculated by combining component standard errors using the variance formula (Ku 1966).

## 3 Results

### 3.1 Variability in Agricultural Management Across Europe

Analysis of farm management data from 248,362 farms revealed that agricultural management practices exhibited significant variability across Europe, both between and within regions. For fertilizer regimes, total N and K inputs showed a similar spatial pattern, with highest average application rates in NW Europe, while total P inputs were highest in northern Italy, NW and NE Europe (Figure S2a–c). The share of N, P, and K from manure followed a similar spatial pattern for all three nutrients, with highest average values in alpine regions and coastal areas of northern Spain (Figure S2d–f). However, the proportion of K derived from manure was consistently higher than that of N or P. Organic farming was most prevalent in marginal regions, that is, with climatic constraints such as in the very north and south or in mountainous regions (Figure S2g). Crop rotational diversity was highest in eastern Germany and Czech Republic (Figure S2h), areas known for large farm sizes. The share of ley and fodder crops in the crop rotation was highest in Sweden, SE France, Portugal, and Italy (Figure S2i). Tillage intensity was generally high, indicating the prevalence of conventional tillage, with lowest values in eastern half of Germany, parts of the UK and France, and southern Portugal (Figure S2j).

While general spatial patterns in farm management across Europe have been described previously (Ballot et al. 2023; Debonne et al. 2022; Helfenstein et al. 2024; Panagos et al. 2022), our study goes beyond existing studies because the use of individual farm data enabled us to describe crop‐ and altitude‐specific management practices for each region, rather than being constrained to regional averages. This is illustrated with the example of Andalusia (region in southern Spain) in Figure S3, where, for example, only 6% of farms growing wheat below 300 m altitude (*n* = 191) were organic, whereas 67% of those with wheat above 600 m (*n* = 89) practiced organic farming.

Of the 257 regions in Europe, our approach identified significant differences in agricultural management in most regions, as illustrated for Andalusia above and in Figure S2. For N, P and K inputs, Kruskal–Wallis tests indicated significant (*p* < 0.05) crop‐ and altitude‐specific differences in 93%, 92%, and 95% of NUTS2 regions, respectively. Significant differences were further observed in 98%, 97% and 97%, respectively of regions for the share of N, P, and K derived from manure; 88% for organic farming, 97% for crop rotational diversity, 95% for the share of ley, 80% for the share of forage, and 89% for tillage intensity. This showcases that our management predictions using individual farms provide more detailed management information than is available from country‐wide or regional averages for a large majority of regions (Ludemann et al. 2024; Panagos et al. 2022).

### 3.2 Permanent Crops and Crops With High Soil Cover Enhance Soil Carbon

**FIGURE 2:**
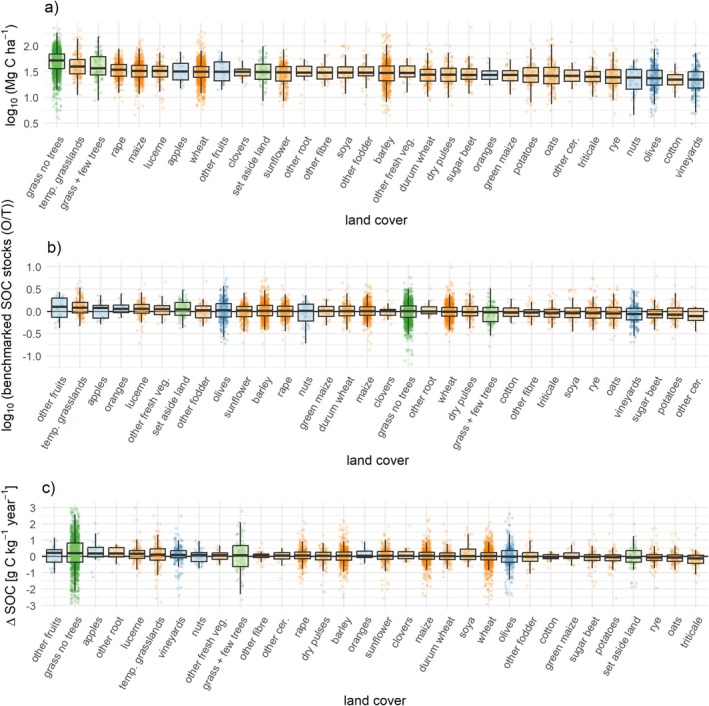
Relationship between soil organic C and land cover. SOC stocks (a, *χ* ^2^ = 1349, *p* < 10^−15^), the benchmarked SOC stocks (b, *χ* ^2^ = 143, *p* < 10^−15^), and the change in soil organic C between 2009 and 2018 (c, *χ* ^2^ = 143, *p* < 10^−15^) were all significantly dependent on land cover. SOC and land cover data from LUCAS Soil. Only land covers with *n* > 15 are shown. Total *n* = 6795, 6795, and 6362 respectively.

SOC stocks (*χ* ^2^ = 1349, *p* < 10^−15^), benchmarked SOC stocks (*χ* ^2^ = 143, *p* < 10^−15^), and the change in SOC concentration between 2009 and 2018 (*χ* ^2^ = 143, *p* < 10^−15^) were all significantly dependent on specific land cover. SOC stocks were highest in grasslands and temporary grasslands (Figure 2a). SOC stocks were calculated using a mass‐corrected fixed depth approach (see method) taking into account variations in concentration of SOC and bulk density. While the concentration of SOC was considerably higher in grasslands than in any arable crops, they also presented lower bulk density than arable crops (Figure S4). Generally, soils under arable cultivation, including temporary grasslands, had significantly higher bulk density than grassland or permanent crops (Figure S4). Some permanent crops showed low observed SOC stocks (Figure 2a,b), as a combination of low bulk density and average to moderate SOC concentration (Figure S4). To account for SOC accumulation potential, observed SOC stocks were benchmarked by dividing by “typical” SOC stocks values for that pedo‐climatic unit (Feeney et al. 2024). Benchmarked SOC stocks were highest in permanent crops and high soil cover arable crops such as temporary grasslands and lucerne, with 70.6% and 63.9% of observations above the benchmark, respectively (Figure 2b). Lowest benchmarked SOC stocks were found in intensive arable crops such as sugar beets (67.1% of observations below benchmark) and potatoes (65.9% of observations below benchmark), which are associated with intensive soil disturbance and minimal crop residue return.

A majority of sites (55%) showed an increase in SOC concentration from 2009 to 2018, confirming De Rosa et al. (2024). Additionally, we found significant differences between individual crops, with most permanent crops and grassland types tending to have lower occurrence of SOC loss (Figure 2c). The crops most likely to have SOC losses were potatoes (58.0%), rye (60.6%), oats (57.5%), and triticale (67%).

### 3.3 Management Intensity Negatively Affects Soil Organic Carbon

**FIGURE 3:**
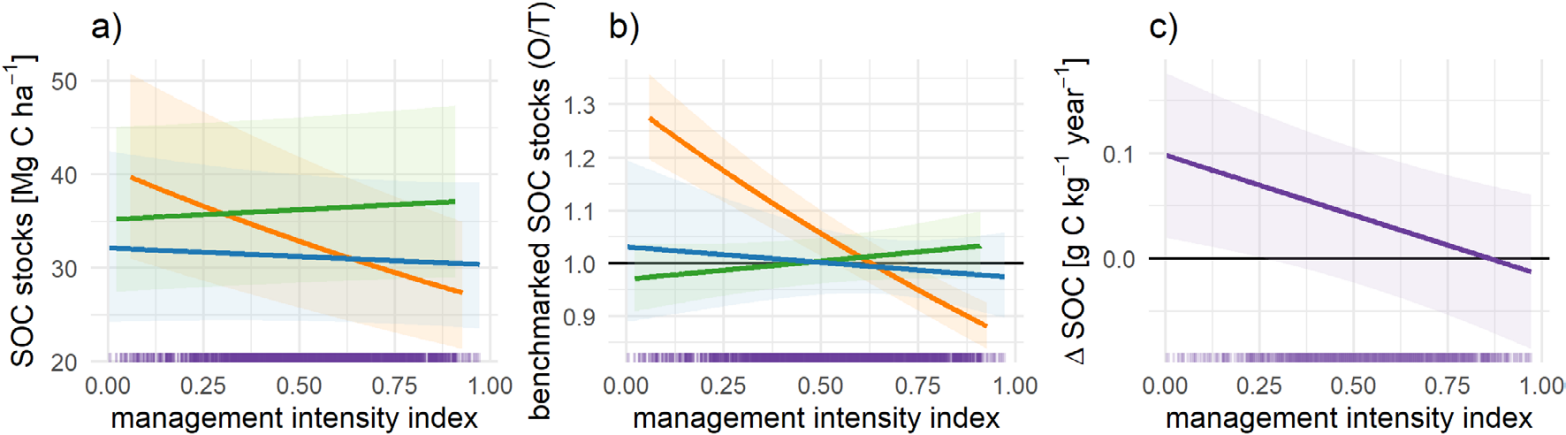
Relationship between SOC and management intensity. Marginal effects calculated from linear mixed‐effects models with pedoclimatic zones (Feeney et al. 2024) as random effect and intensity and land cover class as fixed effect. The model *χ* ^2^ are 95.1 (*p* < 10^−15^) for SOC stocks (a, *n* = 6776), 86.7 (*p* < 10^−15^) for benchmarked SOC stocks (b, *n* = 6776), and 7.3 (*p* = 0.007) for the yearly change in SOC concentration (c, *n* = 6346). See Table S1 for further model details. Orange = arable, blue = permanent crops, green = grassland, and purple = all sites (no significant interaction effect). Shaded areas show the 95% confidence intervals. The black horizontal line in (b) indicates the benchmarked value. Ticks along the x‐axis indicate point density.

Mixed linear models with pedoclimatic zone as a random effect showed that management intensity significantly affected all three SOC metrics (Table S1). Management intensity was calculated from fertilizer input, share of manure, probability of organic cultivation, tillage intensity (arable only), and share of leys and forage in the crop rotation (arable only). SOC metrics in arable sites showed the strongest response to management intensity (Figure 3), with a very clear negative effect of intensity on benchmarked SOC. Arable sites with low management intensities showed a benchmarked SOC of 27% above the pedoclimatic typical values, while the most intensive sites were 11% below (Figure 3b). While management intensity of permanent crops had a slightly negative marginal effect, management intensity of grasslands was positively correlated with both SOC stocks (Figure 3a) and benchmarked SOC stocks (Figure 3b). The rate of change in SOC showed the same negative correlation with intensity index for all three land cover classes (Figure 3c).

**FIGURE 4:**
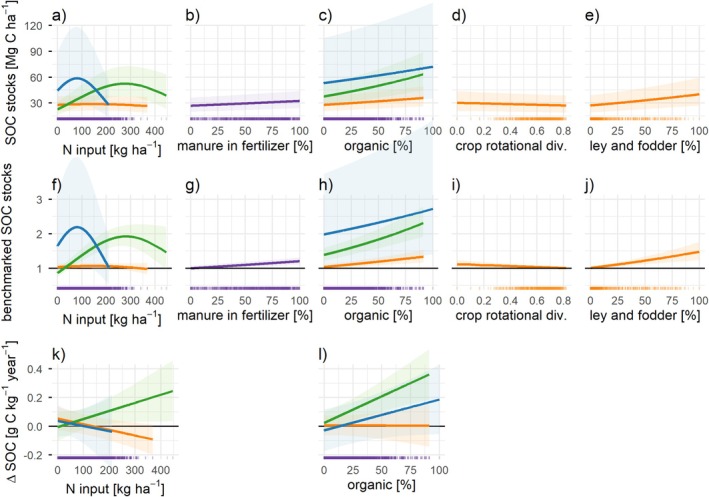
Relationship between SOC and individual management variables. Marginal effects calculated from linear mixed‐effects models with pedoclimatic zones (Feeney et al. 2024) as random effect and management variables as fixed effects. Panels (a–e) show marginal effects on SOC stocks (*n* = 6776), panels (f–j) on benchmarked SOC stocks (*n* = 6776), and panels (k and l) on the change in SOC concentration from 2009 to 2018 (*n* = 6346). Only management variables that had a significant effect are shown, thus some plots are void. Orange = arable, blue = permanent crops, green = grassland, and purple = all sites (no interaction effect). Shaded areas show the 95% confidence intervals. The black horizontal line in (b) indicates the benchmarked value. Crop rotational diversity and the share of ley and fodder crops in the crop rotation only applies to arable crops, thus only orange lines. See Tables S2 and S3 for model details. Ticks along the x‐axis indicate point density.

Regarding the effect of individual management practices, N input had a negative marginal effect on SOC metrics for arable soils, but a positive effect up to 300 kg N ha^−1^ for grassland soils (except for the change in SOC concentration which increased linearly with increasing N inputs) (Figure 4). This observed positive marginal effect of N input on SOC metrics for grasslands explains the positive effect of management intensity overall (Figure 3). The share of manure in the fertilizer regime had a consistently positive marginal effect on SOC stocks and benchmarked SOC stocks (Figure 4b,g). Organic farming had a consistently positive marginal effect on SOC stocks and benchmarked SOC stocks, though with different slopes (Figure 4c,h), but no significant effect on the change in SOC concentration for arable sites (Figure 4l).

The share of ley and fodder crops in the crop rotation had the strongest positive marginal effect on observed and benchmarked SOC stocks in arable soils (Figure 4e,j). A slightly negative effect of crop rotational diversity on SOC stocks (Figure 4d,i) was likely due to rotations with a high share of ley and fodder crops—which tend to have lower diversity. Tillage intensity was not found to have a significant effect on any SOC metric (Table S3), but see data limitations discussed in the Limitations section.

### 3.4 Soil‐ and Climate‐Specific Management Effects

**FIGURE 5:**
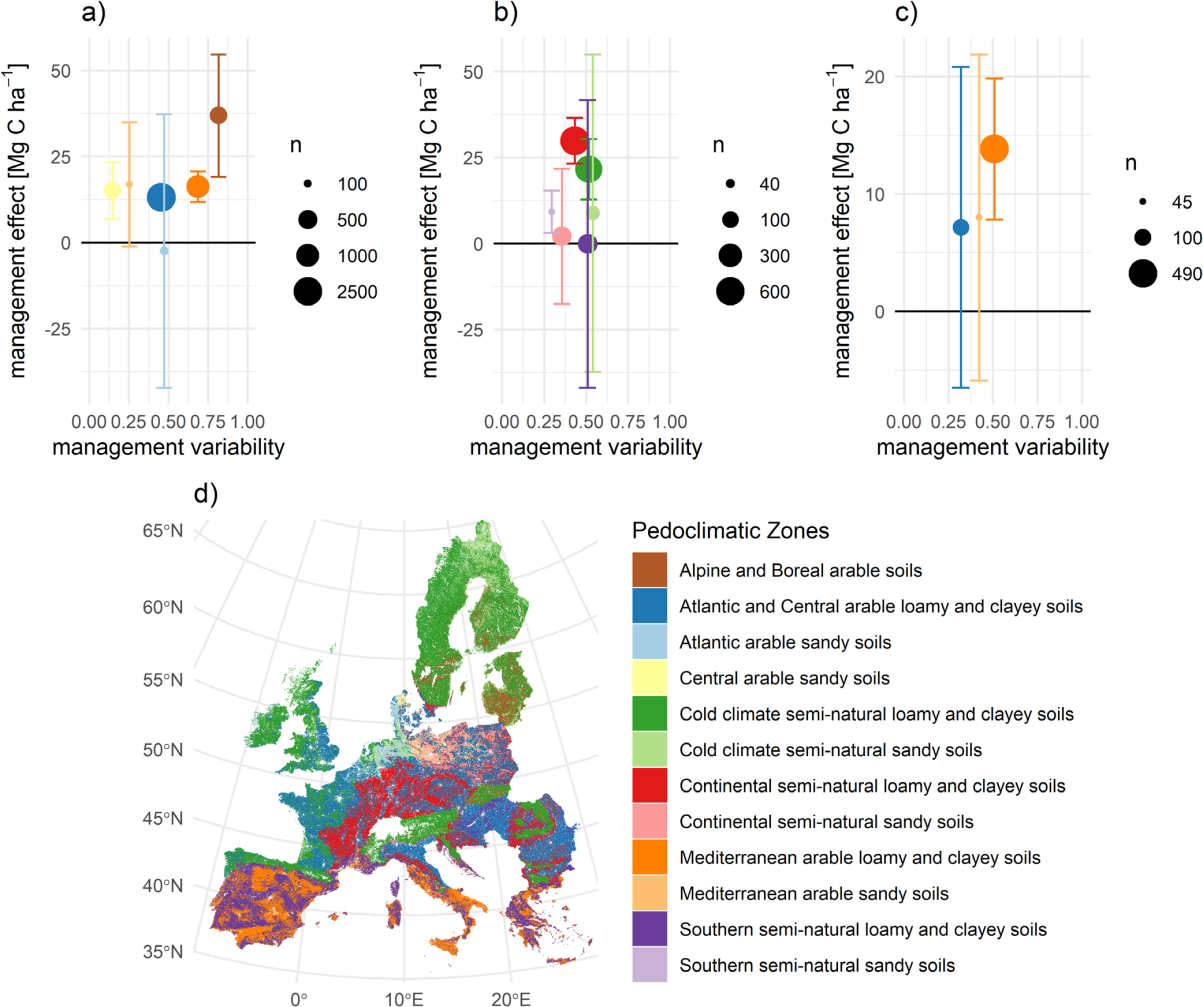
Pedoclimatic zone specific management effect on SOC stocks. (a) Arable, (b) grassland, (c) permanent crop. Management effect is defined as the difference between SOC stocks observed on the 10% most optimally managed and 10% least optimally managed fields per soil pedoclimatic zone. Optimal management is defined as high probability of organic cultivation, high share of manure, low rotational diversity and high share of leys in the rotation for arable; high probability of organic cultivation, high share of manure, and intermediate levels of N input for grassland and permanent crops (see Figure 4). Management variability captures how variable management is within the pedoclimatic zone and is calculated as the mean normalized standard deviation per management indicator. Error bars show the 95% confidence interval. (d) spatial distribution of pedoclimatic zones from Feeney et al. (Feeney et al. 2024).

To test how management effects compared for different pedoclimatic zones, we calculated the difference in observed SOC stocks between the 10% most optimally managed and 10% least optimally managed fields per soil pedoclimatic zone. Optimal management was defined based on results from the mixed linear models (see Figure 5 and methods for details), for example, sites with high values for share of manure in fertilizer mix, organic farming, and share of leys in crop rotation. The strongest effect was observed on Alpine and boreal arable soils, where the difference between 10% most optimally managed sites and 10% least optimally managed sites was 36.9 ± 9.6 Mg C ha^−1^ (Figure 5a and Figure S5). Interestingly, even Central arable sandy soils, for which we observed little management variability, had a significant management effect (Figure 5a), suggesting that these soils are particularly sensitive to management.

For grassland sites, management had a significant effect on SOC stocks in three out of six main pedoclimatic zones (Figure 5b), explaining the most variability (17%) in cold climate loamy and clayey soils (Figure S6). The largest differences between the top 10% managed and bottom 10% managed sites were 29.9 ± 3.4 Mg C ha^−1^ for continental semi‐natural loamy and clayey soils, and 21.6 ± 4.5 Mg C ha^−1^ for cold climate semi‐natural loamy and clayey soils (Figure 5b). For tree crop sites, management explained between 3% and 11% of variability in SOC stocks (Figure S7), and a significant management effect of 13.9 ± 3.1 Mg C ha^−1^ was observed for Mediterranean loamy and clayey soils (Figure 5c), with no consistent effects for the other pedoclimatic zones. Patterns in management effects (Figure 5a–c) aligned well with analysis of observed versus predicted SOC stocks per pedoclimatic zone (Figures S4–S6).

### 3.5 Option Space for Agricultural Practices to Influence Soil Carbon Stocks

Scaling up pedoclimatic zone and crop type specific management effects on SOC stocks (Figure 5) to the European level showed that the option space for agricultural practices to impact SOC stocks ranges from a gain of 1.58 Pg C to a loss of −0.92 Pg C. Under a scenario where all agricultural land is managed according to today's top 10% best managed fields, total SOC stocks could be expected to increase by 1.58 Pg C (95% CI: 1.27–1.89 Pg C). With an increase of 1.12 Pg C (0.86–1.39 Pg C), the largest potential is in arable land, both due to the consistent positive response of SOC stocks to improved management practices across pedoclimatic zones (Figure 5a) and large areas used for arable land (Table S6). Grasslands, with 0.40 Pg C (0.24–0.57 Pg C), and land used for tree crops, with 0.06 Pg C (0.03–0.09 Pg C), had less potential due to lower total area and less consistent management effects. The highest per hectare potential was observed in Alpine and Boreal arable soils.

Under a scenario where all agricultural land is managed according to today's worst 10% management combinations, C loss could be expected to total −0.92 Pg C (−1.15 to −0.68 Pg C). Again, the largest potential loss was calculated in arable soils, with −0.55 Pg C (−0.73 to −0.37 Pg C), followed by grassland with −0.35 Pg C (−0.50 to −0.20 Pg C) and tree crops with −0.02 Pg C (−0.04–0.00 Pg C).

## 4 Discussion

### 4.1 Management Impacts on SOC

While current understanding of how different agricultural practices impact SOC is largely based on analyses (Beillouin et al. 2023; López i Losada et al. 2025; Wiesmeier et al. 2019) and model predictions (Frank et al. 2015; Lugato, Bampa, et al. 2014; Rodrigues et al. 2021) of a limited number of controlled field experiments, our study is the first to leverage large‐scale, real‐farm data to account for the complexity of farming practices and their interactions with diverse soils and climates. This provides both interesting insight on how individual agricultural practices affect SOC and adds to the discussion about total potential SOC gains and or losses under various management scenarios from agricultural practices in Europe.

Other studies using on‐farm data at smaller spatial scales have also found negative correlations between management intensity and SOC in arable soils, for example, in the Netherlands (van Rijssel et al. 2025) or Southern Sweden (Williams et al. 2020), which we show at the continental scale. The negative effect of management intensity highlights the combined effect of various management practices that may deteriorate SOC stocks. A key advantage of our approach is that both the soil and farm management datasets used here were designed to be representative (European Commission 2025a; Orgiazzi et al. 2018), thus capturing the actual variability of soils and management practices at continental scale—an aspect that has been largely absent from previous studies (Edlinger et al. 2023; van Rijssel et al. 2025). The outcomes of our research thus provide crucial empirical evidence to guide effective policy design and practical implementation. For example, our analysis shows that the positive correlation of SOC with organic farming (Bai et al. 2018), share of manure in the fertilizer mix (Beillouin et al. 2023; López i Losada et al. 2025), high crop cover such as through temporary grasslands and other perennial crops (Poeplau and Don 2015; Wiesmeier et al. 2019), and no effect of tillage intensity (Beillouin et al. 2023; Wiesmeier et al. 2019) previously shown for controlled field experiments also holds for real farms across Europe. While controlled experiments have shown that crop rotation positively impacts soil carbon stocks relative to monoculture (Beillouin et al. 2023; McDaniel et al. 2014; Zheng et al. 2023), our study shows that, in practice, the composition of the crop rotation (e.g., high share of leys or forage crops) is more important than diversity of crops per se. This suggests that functional crop diversity, rather than taxonomic diversity alone, is the more relevant mechanism, because rotations that include perennial leys or forage crops can contribute disproportionately to soil carbon accrual through greater root biomass, longer soil cover, and lower disturbance.

Even less is currently known about management impacts on SOC in grasslands and permanent crops (Wiesmeier et al. 2019). Our results show that permanent crops were associated with higher benchmarked SOC stocks compared to arable systems and were more likely to have positive ΔSOC over time (Figure 2), supporting the potential contribution of tree crop systems for increasing SOC stocks (Beillouin et al. 2023; Kay et al. 2019). In terms of sustainable management of grasslands, the positive correlation of fertilizer amount with SOC (Figure 4) adds important nuance to sustainability trade‐offs and context‐dependency of fertilizer use, confirming similar patterns observed at the regional scale on‐farm surveys in England (Ward et al. 2016).

Our study adds nuance to the discussion on SOC sequestration potential of agricultural land by showing that the management effect is highly dependent on soil type (Figure 5), considering two dimensions to site‐specific soil management. The first is that different soils have different potentials for SOC storage (Breure et al. 2025; Georgiou et al. 2025; Poeplau et al. 2024). The second is that different soils respond differently to management. The first has been widely acknowledged through discussions on pedo‐ or soil‐scape zones, or soil districts, by science (Wadoux et al. 2024) and policymakers, for example, in the European Directive on Soil Monitoring and Resilience (Alblas et al. 2025). The second dimension has been emphasized in discussions of context‐specific SOC dynamics (Edlinger et al. 2023; Moinet et al. 2023), but until now has been supported only by piecemeal evidence from limited field experiments or case studies. Our study provides the first continental‐scale empirical demonstration that these relationships can be quantified consistently across diverse regions and agricultural land‐use systems.

### 4.2 Interpreting the Option Space for Agricultural Practices to Impact Soil Carbon

Current SOC stocks in European agricultural soils have been estimated to be 17.6 Pg in the top 30 cm (Lugato, Panagos, et al. 2014). Therefore, our predictions suggest a potential for agricultural management practices, excluding land use change, to improve SOC stocks by 8.9%, or deteriorate them by 5.2%. For proper interpretation of our predictions, it is important to reiterate that they are based on the assumption of immediate, full adoption of the specified “best” or “worst” agricultural practices. These estimates align well with case study evidence, for example, a case study in Austria comparing best‐practice (high rotation diversity, multi‐species cover crop mixtures, minimum tillage and organic fertilization) and conventional farms found that SOC stocks were 14.3 ± 15.5 Mg ha^−1^ or 15.7% ± 18.3% higher in best practice farms (Rosinger et al. 2023).

Under current and planned policy measures across the EU, net emissions are projected to reach a level of 78% below 1990 levels in 2050 (EEA 2025b). This would result in net emissions of an additional 54.1 Pg CO_2_‐eq by 2050, or 58.6 Pg CO_2_ (equivalent to 16.0 Pg C) excluding removals from the Land Use, Land Use Change and Forestry sector (EEA 2025a). Assuming immediate and full deployment of best farm management practices across Europe and that a new SOC steady‐state is reached over 25 years, our estimates suggest that soils could offset about 10% of these emissions. Inversely European agricultural soils, if poorly managed, could increase cumulative emissions to 2050 by up to 6%. If European emissions remain at their 2050 projected level (1.5 Pg CO_2_‐eq per year) (EEA 2025b) to the end of the century, cumulative emissions would decrease the proportional contribution of soil to 4%.

These estimates align with previous studies, while being slightly more optimistic. Predictions with the CENTURY model allocating 12% to 28% of the European arable land to alternative management practices resulted in a potential SOC sequestration of 549–2141 Mt. CO_2_ eq. by 2100, equating to 0.15 to 0.58 Pg C (Lugato, Bampa, et al. 2014). While our estimates are higher, it has to be noted that we considered not only adoption of best practices on a fraction of arable but almost all agricultural land, and our estimates are based on empirical farm data while previous studies have used model simulations or national statistics. However, the aforementioned study also considered more invasive changes such as land use conversion from arable to grassland. Another study estimated 15.2 Tg C year^−1^ for the sum of country level potential SOC sequestration rates in European agricultural soils (Rodrigues et al. 2021).

It is important to note that SOC sequestration potential is finite (Moinet et al. 2023; Smith 2008), and grows smaller in proportion as projected cumulative emissions increase. Inversely, emissions are not finite so long as fossil energy can be extracted and would have the potential to continue well beyond 2050. Also, given the finite and reversible nature of SOC sequestration, its primary role should be seen in supporting soil health, with climate mitigation as a valuable co‐benefit (Moinet et al. 2023).

## 5 Limitations

We identify and discuss three main limitations of our study. The first is that our predictions of management do not fully capture actual management at every point location. Nevertheless, by using detailed data from > 250,000 individual farms, our approach provides a more nuanced representation of management than previously available datasets. Our analyses further show that, given a sufficiently large sample size, this method can reliably detect underlying management effects. However, since tillage was not available in the individual farm dataset, aggregated data from EUROSTAT (2016) was used for this variable (EUROSTAT 2020). Hence, tillage data in this study has poorer resolution than the other management variables. It is plausible that this temporal and spatial aggregation attenuates the detected signal of tillage effects, particularly in regions with high variability in tillage practices between crops or with rapid adoption of conservation tillage post‐2016.

Second, predictions of the option space are made using simple scenarios based on absolute differences in C stocks. This represents the total or potential carbon that could be offset if all were managed like the top 10%, not accounting for time and assuming no land use conversion. Our estimates also do not account for environmental change; for example, through climate change, which has been described previously (Smith et al. 2005).

Thirdly, while ΔSOC is potentially the most interesting SOC indicator from a monitoring perspective, the noise to signal ratio for ΔSOC was particularly high, which is a challenge faced by all SOC monitoring studies. This becomes apparent when juxtaposing observed yearly ΔSOC rates with typical values of measurement errors. While observed ΔSOC were on the range of ±0.1 g kg^−1^ year^−1^ (Figure 2), ranges also reported by other studies (Beillouin et al. 2023; De Rosa et al. 2024), the relative error in SOC measurement has been shown to be 22.1% using data from laboratory proficiency testing schemes (van Leeuwen et al. 2022), that is, ±3.3 g SOC kg^−1^ for a typical cropland soil with 15 g SOC kg^−1^. This means measurement errors are more than an order of magnitude higher than expected management effects, presenting a considerable challenge for monitoring change of SOC, for example, for C certificates. Three ways to overcome this dilemma are measuring ΔSOC over long time spans, with large sample sizes, or by using a model of SOC stocks with quantified uncertainty at each time step, which can then be used to infer ΔSOC and its variance. It is also critical to stress that monitoring SOC, particularly for climate mitigation outcomes, should include assessment of bulk density, which is itself sensitive to management (Figure S4) and whose variation can prevent reliable deduction of changes in the quantity of C in the soil (Moinet et al. 2023).

## 6 Outlook

While management practices have largely been overlooked in previous large‐scale SOC monitoring studies (Breure et al. 2025; De Rosa et al. 2024; Levy et al. 2024; Xie et al. 2007), our study addresses this critical gap and provides a blueprint for future studies assessing impacts of agricultural practices on SOC or other soil health indicators. Globally, soil monitoring initiatives are expanding rapidly, with major programs underway or planned in Europe (Alblas et al. 2025), China (The State Council of the People's Republic of China 2024), India (Reddy 2019), Africa (Home | Soils 4 Africa—Horizon 2020 Programme of the European Union 2025), and Australia (Australian Government—DAFF 2025). Our results demonstrate that routinely reported farm‐record data are powerful predictors of SOC dynamics, even without plot‐specific management histories. This approach revealed that the option space for agricultural practices to increase/decrease SOC stocks varies by pedoclimatic zones. To optimize SOC management, future policy should (1) avoid blanket approaches that apply generic guidance across regions, instead tailoring strategies to local soil and socio‐economic conditions, and (2) ensure that management data are systematically integrated into soil monitoring schemes. Future soil monitoring programs should therefore be designed from the outset to enable linkage with farm management record systems, for example through the collection of standardized, simplified management histories at soil sampling locations, while safeguarding the privacy of individual land users. In combination with farm management surveys, large scale soil monitoring can play a crucial role as we move forward to assess and balance trade‐offs of various agricultural practices on soil functions across environmental gradients.

## Author Contributions

**Anna Edlinger:** conceptualization, writing – review and editing, methodology. **Gabriel Y. K. Moinet:** investigation, writing – review and editing, conceptualization. **Rachel Creamer:** writing – review and editing. **Carmen Vazquez:** writing – review and editing. **Julian Helfenstein:** conceptualization, investigation, writing – original draft, writing – review and editing, methodology, supervision. **Nick van Dijk:** investigation, methodology, writing – review and editing. **Vera L. Mulder:** conceptualization, writing – review and editing. **Sophie Q. van Rijssel:** supervision, methodology, writing – review and editing. **Alexandre M. J. – C. Wadoux:** investigation, methodology, writing – review and editing.

## Funding

This work was supported by the Dutch Ministry of Education: Earth and Environmental Sciences sector plan (JH, SvR) and the European Union's Horizon Europe research and Innovation program under Grant Agreement: 101091010, Project BENCHMARKS (RC, CV, VLM).

## Disclosure

*Artificial Intelligence Generated Content*: Authors used ChatGPT to improve legibility of individual sentences. After using this tool, the authors reviewed and edited the affected sentences. AI was not used to produce content, and the authors take full responsibility for the content of the publication.

## Conflicts of Interest

The authors declare no conflicts of interest.

## Supporting information


**Figure S1:** Comparison between measured fine soil bulk density from LUCAS 2018 campaign and predicted fine soil bulk density (Bd, ref.) using edaphic properties as detailed in the main manuscript. The 1:1 line is represented as a dashed line. Color gradient represents the number of observations for specific bulk densities.
**Figure S2:** Spatial variability in farm management based on 81,688 representative individual farm observations from 2018. Maps show the area‐weighted mean value of a farm management indicator per NUTS2 administrative region. (a–c) total N, P, K input in kg ha^−1^, including both mineral fertilizer and manure; (d–f) the share of N, P, K respectively derived from manure; (g) is the prevalence of organic farming (% of area); (h) rotational diversity measured as the Gini‐Simpson index–score close to one means high diversity; (i) share of ley and fodder legumes in the crop mix; (j) tillage intensity, see methods for details. See Figure S1 for the number of farms (sample size) in each NUTS2 region.
**Figure S3:** Variability of management practices within a region. Andalusia, southernmost region in Spain, is used as an example to illustrate crop and altitude specific management calculated for all NUTS2 regions in the EU. Error bars in the second column show the standard error of area‐weighted medians. Sample size varies from *n* = 23 (potatoes at 300–600 m above sea level) to 714 (cotton at < 300 m above sea level) farm observations per point.
**Figure S4:** Relationship between soil organic C indicators and land cover. SOC concentration (a, *χ* ^2^ = 2475.5, *p* < 10^−15^) and soil bulk density (b, *χ* ^2^ = 455.1, *p* < 10^−15^) were significantly dependent on land cover. Only land covers with *n* > 14 are shown. Total *n* = 8760 and 2809 respectively.
**Figure S5:** Pedoclimatic zone specific management effect for arable soils. Observations versus predictions based on the linear mixed effects models show that the correlation between management and SOC stocks is stronger in some pedoclimatic zones than others. Sample size is 392 for Alpine and Boreal soils, 2583 for Atlantic and Central loamy and clayey soils, 105 for Atlantic sandy soils, 379 for Central sandy soils, 1003 for Mediterranean loamy and clayey soils, and 97 for Mediterranean sandy soils.
**Figure S6:** Pedoclimatic zone specific management effect for grassland soils. Observations versus predictions based on the linear mixed effects models show that the correlation between management and SOC stocks is stronger in some pedoclimatic zones than others. Only relationships for the most common (*n* > 30) pedoclimatic zones are shown. Sample size is 496 for Cold climate loamy and clayey soils, 69 for Cold climate sandy soils, 637 for Continental loamy and clayey soils, 152 for Continental sandy soils, 162 for Southern loamy and clayey soils, and 37 for Southern sandy soils.
**Figure S7:** Pedoclimatic zone specific management effect for tree soils. Observations versus predictions based on the linear mixed effects models show that the correlation between management and SOC stocks is stronger in some pedoclimatic zones than others. Only relationships for the most common (*n* > 30) pedoclimatic zones are shown. Sample size is 96 for Atlantic and Central loamy and clayey soils, 497 for Mediterranean loamy and clayey soils, and 45 for Mediterranean sandy soils.
**Table S1:** Linear mixed model to analyze relationship between SOC indicators and management intensity. Marginal effects are shown in Figure 2 of the main text. The model χ^2^ are 95.1 (*p* < 10^−15^) for SOC stocks (*n* = 6776), 86.7 (*p* < 10^−15^) for benchmarked SOC stocks (*n* = 6776), and 7.3 (*p* = 0.007) for the yearly change in SOC (ΔSOC) concentration (*n* = 6346).
**Table S2:** Linear mixed model to analyze relationships between SOC indicators and management indicators. Marginal effects are shown in Figure 3 of the main text. The model *χ* ^2^ are 430.4 (*p* < 10^−15^) for SOC stocks (*n* = 6776), 410.0 (*p* < 10^−15^) for benchmarked SOC stocks (*n* = 6776), and 30.4 (*p* ≤ 0.001) for the yearly change in SOC (ΔSOC) concentration (*n* = 6346).
**Table S3:** Linear mixed model to analyze relationships between SOC indicators and management indicators, arable only. In comparison to models from table 2, these models were fit to arable sites only, using also management indicators for arable farming, namely crop rotation diversity, tillage, and share of ley/forage. Marginal effects are shown in Figure 3 of the main text. The model *χ* ^2^ are 250.5 (*p* < 10^−15^) for SOC stocks (*n* = 4504), 249.4 (*p* < 10^−15^) for benchmarked SOC stocks (*n* = 4504), and 62.3 (*p* ≤ 0.001) for the yearly change in SOC (ΔSOC) concentration (*n* = 4326).
**Table S4:** Covariate data and sources.
**Table S5:** Areas per land cover and pedoclimatic zone.
**Table S6:** SOC stocks per pedoclimatic zone and option space for gains/losses. Q10 refers to the points with the 10% worst management in terms of SOC stocks, and Q90 to the best 10%. The “worst scenario” refers to a scenario whereby all agricultural land is cultivated according to the current worst 10%, and “best scenario” refers to the scenario whereby all agricultural land is cultivated according to the current best 10%. Standard error of the sum (bottom of table) is calculated using the variance formula (Ku et al., 1966).

## Data Availability

LUCAS Soil survey data used in this study was made available by the European Commission through the European Soil Data Centre managed by the Joint Research Center. It is publicly available in https://esdac.jrc.ec.europa.eu/content/lucas‐2009‐topsoil‐data for 2009 and https://esdac.jrc.ec.europa.eu/content/lucas‐2018‐topsoil‐data for 2018. Farm management data was provided by D‐Agri of the European Commission (data access contract IFD 2023_06) and cannot be shared publicly due to the sensitive nature of this data. For details and references of further publicly available datasets used in the study see Table S4.
