# Supplementary material for: Continental‐Scale Evidence of Farm Management Impacts on Soil Carbon
Source: Glob Chang Biol. 2026 May 15;32:e70913. doi: 10.1111/gcb.70913 (PMC13178209; doi:10.1111/gcb.70913)
Supplement: Supplementary file 1 — Figure S1: Comparison between measured fine soil bulk density from LUCAS 2018 campaign and predicted fine soil bulk density (Bd, ref.) using edaphic properties as detailed in the main manuscript. The 1:1 line is represented as a dashed line. Color gradient represents the number of observations for specific bulk densities. Figure S2: Spatial variability in farm management based on 81,688 representative individual farm observations from 2018. Maps show the area‐weighted mean value of a farm management indicator per NUTS2 administrative region. (a–c) total N, P, K input in kg ha−1, including both mineral fertilizer and manure; (d–f) the share of N, P, K respectively derived from manure; (g) is the prevalence of organic farming (% of area); (h) rotational diversity measured as the Gini‐Simpson index–score close to one means high diversity; (i) share of ley and fodder legumes in the crop mix; (j) tillage intensity, see methods for details. See Figure S1 for the number of farms (sample size) in each NUTS2 region. Figure S3: Variability of management practices within a region. Andalusia, southernmost region in Spain, is used as an example to illustrate crop and altitude specific management calculated for all NUTS2 regions in the EU. Error bars in the second column show the standard error of area‐weighted medians. Sample size varies from n = 23 (potatoes at 300–600 m above sea level) to 714 (cotton at < 300 m above sea level) farm observations per point. Figure S4: Relationship between soil organic C indicators and land cover. SOC concentration (a, χ 2 = 2475.5, p < 10−15) and soil bulk density (b, χ 2 = 455.1, p < 10−15) were significantly dependent on land cover. Only land covers with n > 14 are shown. Total n = 8760 and 2809 respectively. Figure S5: Pedoclimatic zone specific management effect for arable soils. Observations versus predictions based on the linear mixed effects models show that the correlation between management and SOC stocks is stronger in some pedoclima [file GCB-32-e70913-s001.docx]

Supplementary Materials for

**Continental-scale evidence of farm management impacts on soil carbon**

Julian Helfenstein et al.

*Corresponding author. Email: [julian.helfenstein@wur.nl](mailto:julian.helfenstein@wur.nl)

**This PDF file includes:**

Figs. S1 to S7

Tables S1 to S5

References

Supplementary figures


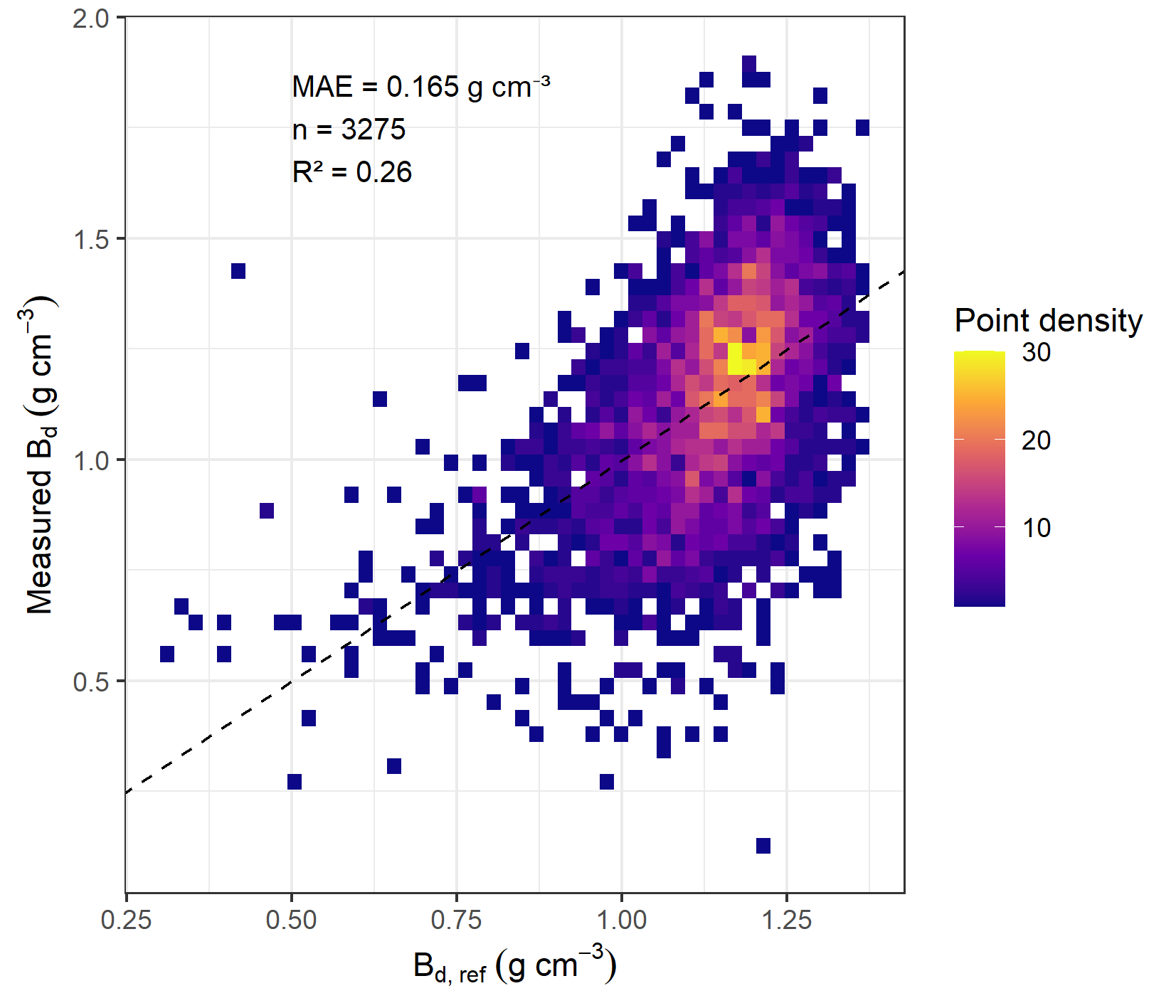


Fig. S1. Comparison between measured fine soil bulk density from LUCAS 2018 campaign and predicted fine soil bulk density (Bd, ref¬) using edaphic properties as detailed in the main manuscript. The 1:1 line is represented as a dashed line. Color gradient represents the number of observations for specific bulk densities.


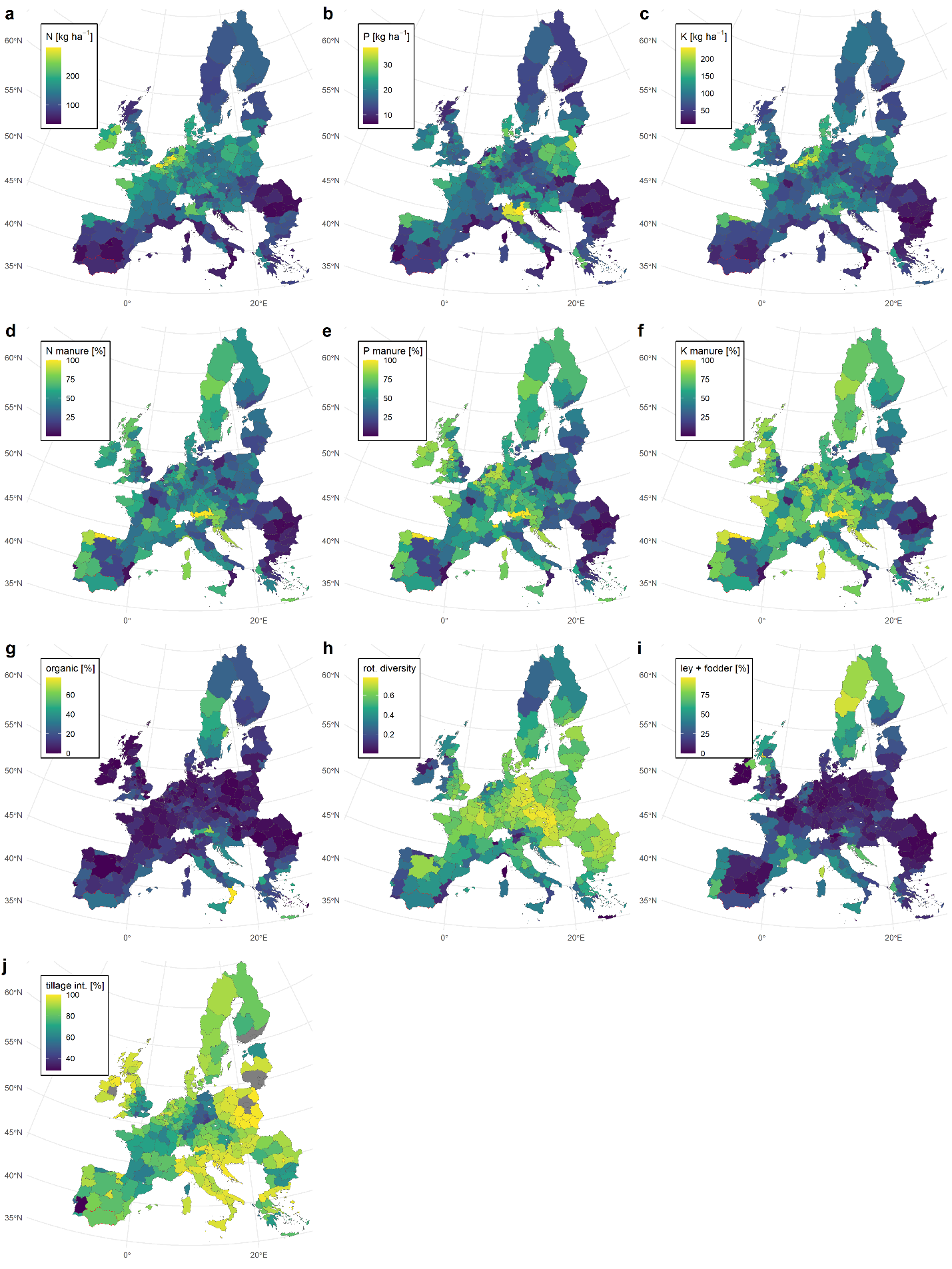


**Figure S2. Spatial variability in farm management based on 81,688 representative individual farm observations from 2018.** Maps show the area-weighted mean value of a farm management indicator per NUTS2 administrative region. a) – c) total N, P, K input in kg ha^-1^, including both mineral fertilizer and manure; d) – f) the share of N, P, K respectively derived from manure; g) is the prevalence of organic farming (% of area); h) rotational diversity measured as the Gini-Simpson index –score close to one means high diversity; i) share of ley and fodder legumes in the crop mix; j) tillage intensity, see methods for details. See Supplementary Fig. 1 for the number of farms (sample size) in each NUTS2 region.


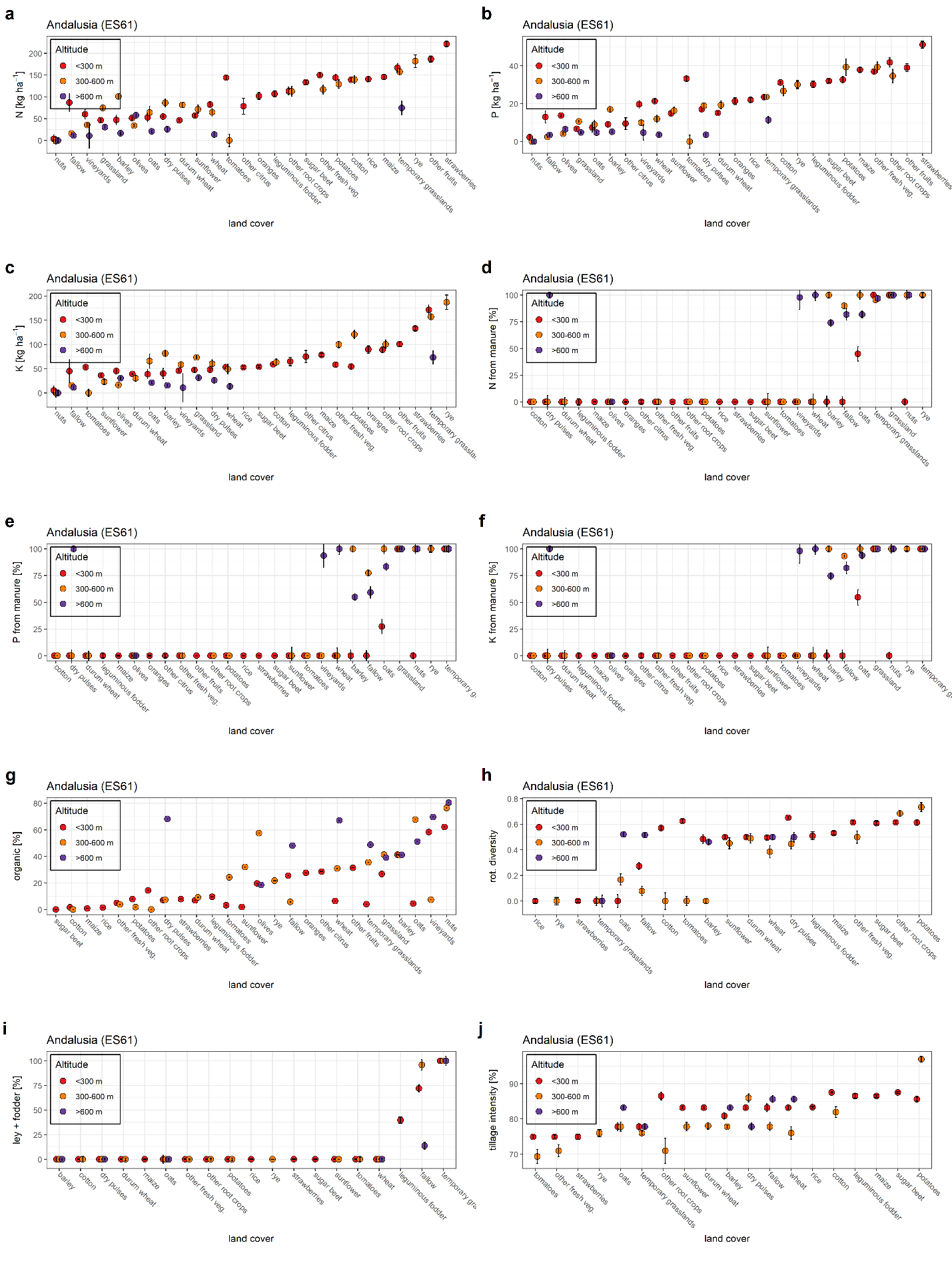


**Figure S3. Variability of management practices within a region.** Andalusia, southernmost region in Spain, is used as an example to illustrate crop and altitude specific management calculated for all NUTS2 regions in the EU. Error bars in the second column show the standard error of area-weighted medians. Sample size varies from n = 23 (potatoes at 300 – 600 m above sea level) to 714 (cotton at < 300 m above sea level) farm observations per point.


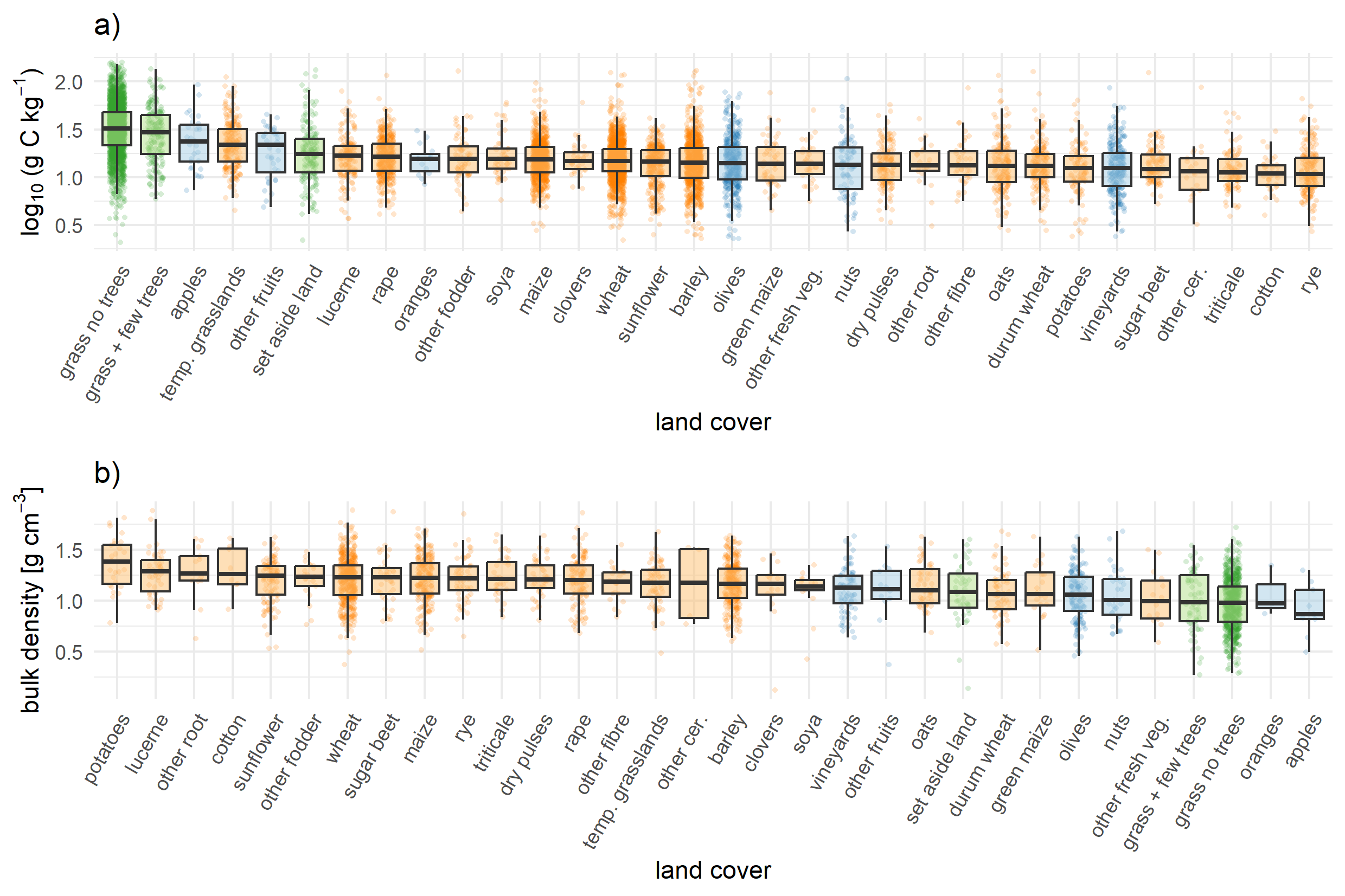


Figure S4. Relationship between soil organic C indicators and land cover. SOC concentration (a), χ² = 2475.5, p < 10^-15^) and soil bulk density (b), χ² = 455.1, p < 10^-15^) were significantly dependent on land cover. Only land covers with n > 14 are shown. Total n = 8,760 and 2,809 respectively.


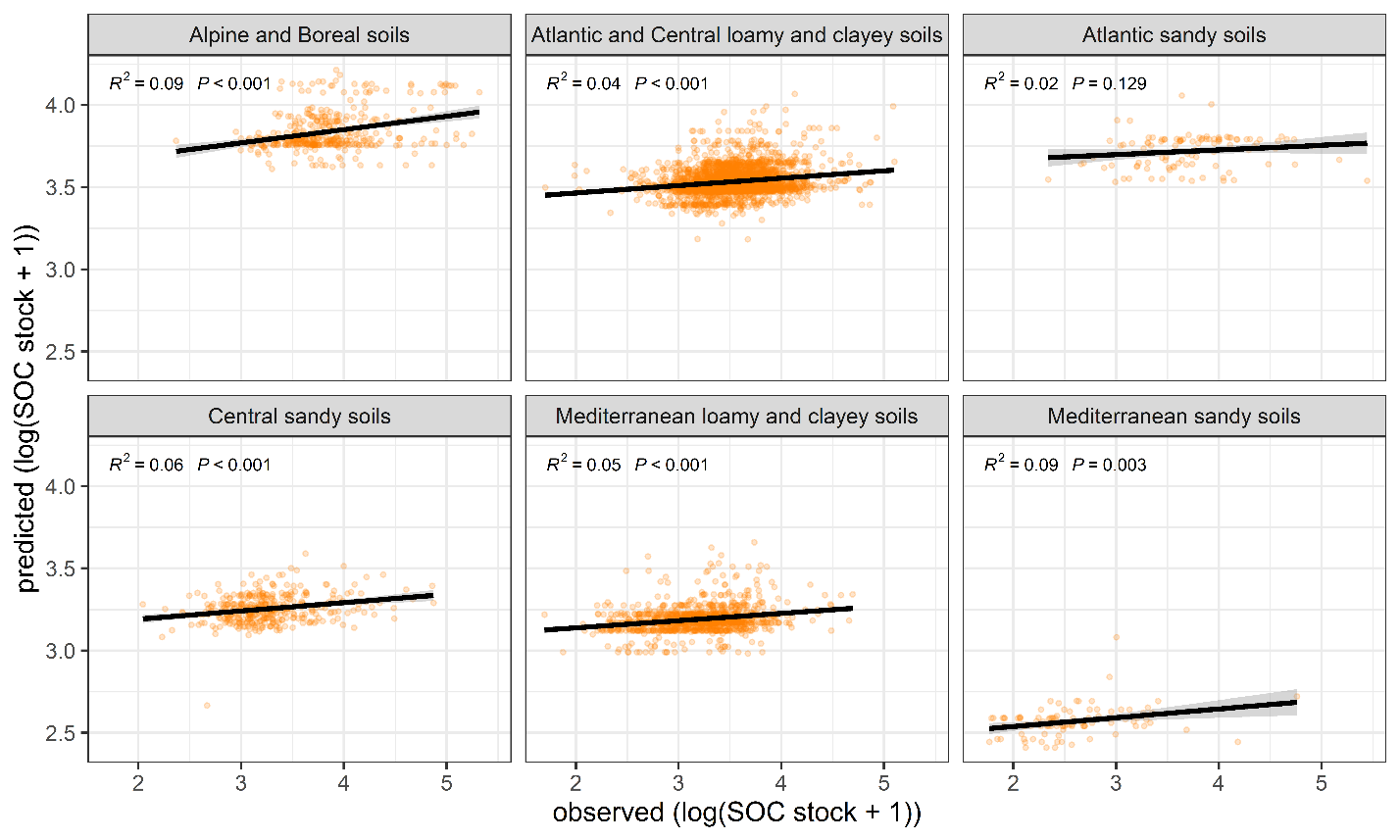


Figure S5. Pedoclimatic zone specific management effect for arable soils. Observations versus predictions based on the linear mixed effects models show that the correlation between management and SOC stocks is stronger in some pedoclimatic zones than others. Sample size is 392 for Alpine and Boreal soils, 2583 for Atlantic and Central loamy and clayey soils, 105 for Atlantic sandy soils, 379 for Central sandy soils, 1003 for Mediterranean loamy and clayey soils, and 97 for Mediterranean sandy soils.


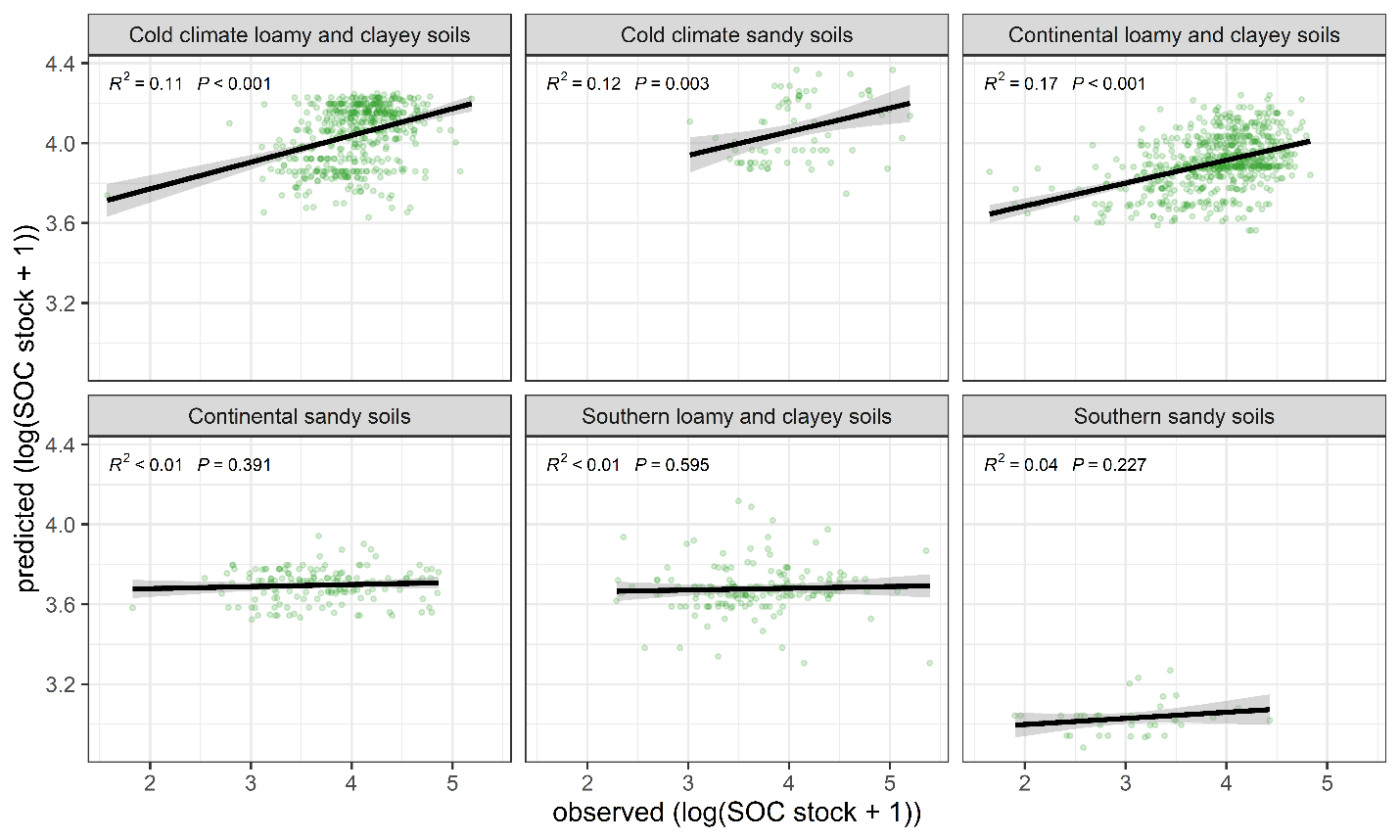


Figure S6. Pedoclimatic zone specific management effect for grassland soils. Observations versus predictions based on the linear mixed effects models show that the correlation between management and SOC stocks is stronger in some pedoclimatic zones than others. Only relationships for the most common (n > 30) pedoclimatic zones are shown. Sample size is 496 for Cold climate loamy and clayey soils, 69 for Cold climate sandy soils, 637 for Continental loamy and clayey soils, 152 for Continental sandy soils, 162 for Southern loamy and clayey soils, and 37 for Southern sandy soils.


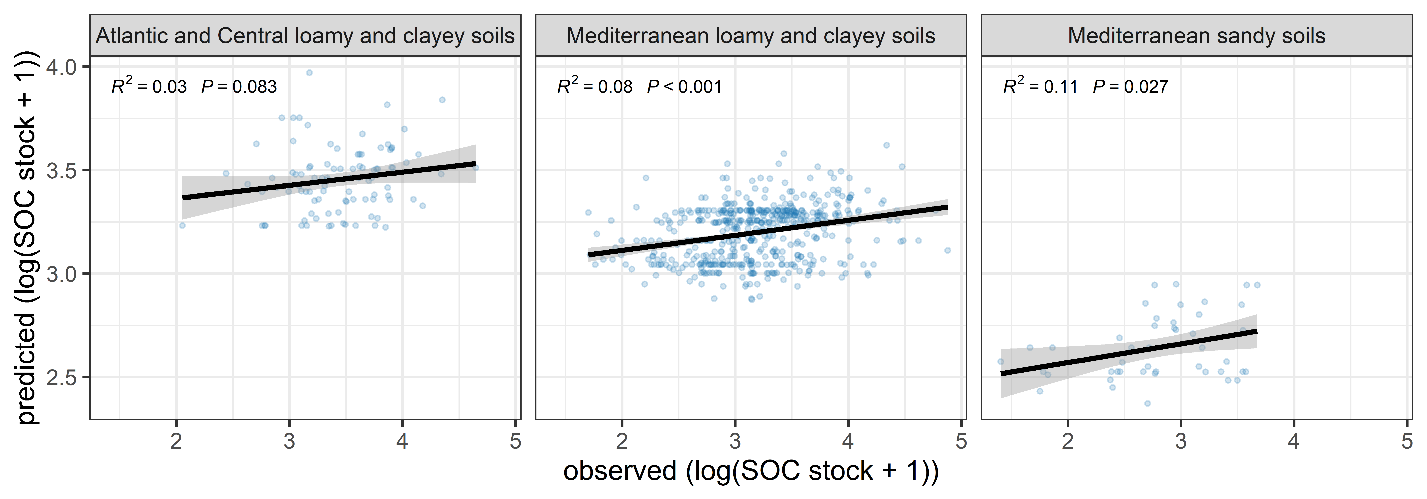


Figure S7. Pedoclimatic zone specific management effect for tree soils. Observations versus predictions based on the linear mixed effects models show that the correlation between management and SOC stocks is stronger in some pedoclimatic zones than others. Only relationships for the most common (n > 30) pedoclimatic zones are shown. Sample size is 96 for Atlantic and Central loamy and clayey soils, 497 for Mediterranean loamy and clayey soils, and 45 for Mediterranean sandy soils.

Supplementary tables

**Table S1. Linear mixed model to analyze relationship between SOC indicators and management intensity.** Marginal effects are shown in Fig. 2 of the main text. The model χ² are 95.1 (p < 10^-15^) for SOC stocks (n = 6,776), 86.7 (p < 10^-15^) for benchmarked SOC stocks (n = 6,776), and 7.3 (p = 0.007) for the yearly change in SOC (ΔSOC) concentration (n = 6,346).

|  |  |  | SOC stocks | |  | benchmarked stocks | |  | ΔSOC | |
| --- | --- | --- | --- | --- | --- | --- | --- | --- | --- | --- |
| variable | DoF |  | F-value | p |  | F-value | p |  | F-value | p |
| intensity | 1 |  | 11.40 | < 0.001 |  | 10.67 | 0.001 |  | 7.28 | 0.007 |
| land cover group | 2 |  | 5.82 | 0.003 |  | 20.55 | < 0.001 |  |  |  |
| intensity*(land cover group) | 2 |  | 24.19 | < 0.001 |  | 25.20 | < 0.001 |  |  |  |

**Table S2. Linear mixed model to analyze relationships between SOC indicators and management indicators.** Marginal effects are shown in Fig. 3 of the main text. The model χ² are 430.4 (p < 10^-15^) for SOC stocks (n = 6,776), 410.0 (p < 10^-15^) for benchmarked SOC stocks (n = 6,776), and 30.4 (p = < 0.001) for the yearly change in SOC (ΔSOC) concentration (n = 6,346).

|  |  |  | SOC stocks | |  | benchmarked stocks | |  | ΔSOC | |
| --- | --- | --- | --- | --- | --- | --- | --- | --- | --- | --- |
| variable | DoF |  | F-value | p |  | F-value | p |  | F-value | p |
| land cover | 39 |  | 2.47 | < 0.001 |  | 3.14 | < 0.001 |  |  |  |
| land cover group | 2 |  | 15.61 | < 0.001 |  | 14.64 | < 0.001 |  | 0.68 | 0.510 |
| N fertilizer | 1 |  | 0.11 | 0.741 |  | 0.03 | 0.866 |  | 0.15 | 0.694 |
| (N fertilizer)^2 | 1 |  | 21.36 | < 0.001 |  | 21.22 | < 0.001 |  |  |  |
| N fertilizer*land cover group | 2 |  | 49.63 | < 0.001 |  | 45.45 | < 0.001 |  | 3.55 | 0.029 |
| (N fertilizer)^2*land cover group | 2 |  | 13.38 | < 0.001 |  | 12.53 | < 0.001 |  |  |  |
| manure | 1 |  | 15.88 | < 0.001 |  | 15.59 | < 0.001 |  |  |  |
| manure*land cover group | 2 |  |  |  |  |  |  |  |  |  |
| organic | 1 |  | 67.54 | < 0.001 |  | 65.22 | < 0.001 |  | 11.52 | < 0.001 |
| organic*land cover group | 2 |  | 9.69 | < 0.001 |  | 8.74 | < 0.001 |  | 5.05 | 0.007 |

**Table S3. Linear mixed model to analyze relationships between SOC indicators and management indicators, arable only**. In comparison to models from table 2, these models were fit to arable sites only, using also management indicators for arable farming, namely crop rotation diversity, tillage, and share of ley/forage. Marginal effects are shown in Fig. 3 of the main text. The model χ² are 250.5 (p < 10^-15^) for SOC stocks ( n = 4,504), 249.4 (p < 10^-15^) for benchmarked SOC stocks (n = 4,504), and 62.3 (p = < 0.001) for the yearly change in SOC (ΔSOC) concentration (n = 4,326).

|  |  |  | SOC stocks | |  | benchmarked stocks | |  | ΔSOC | |
| --- | --- | --- | --- | --- | --- | --- | --- | --- | --- | --- |
| variable | DoF |  | F-value | p |  | F-value | p |  | F-value | p |
| land cover | 28 |  | 3.23 | < 0.001 |  | 3.13 | < 0.001 |  | 2.26 | < 0.001 |
| N fertilizer | 1 |  |  |  |  |  |  |  |  |  |
| (N fertilizer)^2 | 1 |  |  |  |  |  |  |  |  |  |
| manure | 1 |  | 4.19 | 0.041 |  | 5.35 | 0.021 |  | 9.72 | 0.002 |
| organic | 1 |  | 13.67 | < 0.001 |  | 13.54 | < 0.001 |  |  |  |
| tillage | 1 |  |  |  |  |  |  |  |  |  |
| rotational diversity | 1 |  | 10.04 | 0.002 |  | 9.55 | 0.002 |  |  |  |
| ley_forage | 1 |  | 64.97 | < 0.001 |  | 62.64 | < 0.001 |  |  |  |

**Table S4. Covariate data and sources**.

| **variable** | **unit** | **definition** | **source** |
| --- | --- | --- | --- |
| MAT | °C | mean annual temperature | WorldClim ^2^ |
| MAP | mm | mean annual precipitation | WorldClim ^2^ |
| aridity | - | aridity index | Global aridity ^3^ |
| ET0 | mm | potential evapotranspiration | Global potential evapotranspiration ^3^ |
| Koppen | categorical | Köppen–Geiger climate classification | Global climate classification ^4^ |
| elevation | m | meters above sea level | LUCAS Soil |
| WRB | categorical | World Reference Base soil groups | LUCAS Soil |
| parent_material | categorical | main lithological units | LUCAS Soil |
| other soil properties | various | clay, silt and sand content [%]; oxalate-extractable Fe and Al concentration [g kg^-1^]; pH measured in water; electrical conductivity [millisievert]; availalbe P (Olsen's) [mg kg^-1^]; exchangeable K [mg kg^-1^] | LUCAS Soil |

Table S5. Areas per land cover and pedoclimatic zone.

| **Land Cover** | **Pedoclimatic Zone** | **Area (thousand km^2^)** |
| --- | --- | --- |
| Cropland | Alpine and Boreal arable soils | 85.9 |
|  | Atlantic and Central arable loamy and clayey soils | 718.5 |
|  | Atlantic arable sandy soils | 25.4 |
|  | Central arable sandy soils | 34.3 |
|  | Cold climate semi-natural loamy and clayey soils | 41.5 |
|  | Cold climate semi-natural sandy soils | 4.6 |
|  | Continental semi-natural loamy and clayey soils | 48.6 |
|  | Continental semi-natural sandy soils | 8.4 |
|  | Mediterranean arable loamy and clayey soils | 225.9 |
|  | Mediterranean arable sandy soils | 0.0 |
|  | Southern semi-natural loamy and clayey soils | 44.0 |
|  | Southern semi-natural sandy soils | 0.0 |
|  | Total cropland: | 1237.0 |
| Grassland | Alpine and Boreal arable soils | 28.2 |
|  | Atlantic and Central arable loamy and clayey soils | 129.6 |
|  | Atlantic arable sandy soils | 4.5 |
|  | Central arable sandy soils | 3.8 |
|  | Cold climate semi-natural loamy and clayey soils | 179.0 |
|  | Cold climate semi-natural sandy soils | 9.1 |
|  | Continental semi-natural loamy and clayey soils | 115.3 |
|  | Continental semi-natural sandy soils | 10.1 |
|  | Mediterranean arable loamy and clayey soils | 8.2 |
|  | Mediterranean arable sandy soils | 0.0 |
|  | Southern semi-natural loamy and clayey soils | 28.0 |
|  | Southern semi-natural sandy soils | 0.0 |
|  | Total grassland: | 515.9 |
| Tree crops | Alpine and Boreal arable soils | 0.0 |
|  | Atlantic and Central arable loamy and clayey soils | 0.8 |
|  | Atlantic arable sandy soils | 0.0 |
|  | Central arable sandy soils | 0.0 |
|  | Cold climate semi-natural loamy and clayey soils | 0.1 |
|  | Cold climate semi-natural sandy soils | 0.0 |
|  | Continental semi-natural loamy and clayey soils | 0.1 |
|  | Continental semi-natural sandy soils | 0.0 |
|  | Mediterranean arable loamy and clayey soils | 52.6 |
|  | Mediterranean arable sandy soils | 0.0 |
|  | Southern semi-natural loamy and clayey soils | 6.6 |
|  | Southern semi-natural sandy soils | 0.0 |
|  | Total tree crops: | 60.3 |

Table S6. SOC stocks per pedoclimatic zone and option space for gains/losses. Q10 refers to the points with the 10% worst management in terms of SOC stocks, and Q90 to the best 10%. The “worst scenario” refers to a scenario whereby all agricultural land is cultivated according to the current worst 10%, and “best scenario” refers to the scenario whereby all agricultural land is cultivated according to the current best 10%. Standard error of the sum (bottom of table) is calculated using the variance formula (Ku et al., 1966).

|  |  |  |  | point values | | | | | | |  | worst scenario | |  | best scenario | |
| --- | --- | --- | --- | --- | --- | --- | --- | --- | --- | --- | --- | --- | --- | --- | --- | --- |
| LC | pedoclimatic zone | area [thousand ha] |  | mean SOC [Mg ha^-1^] | se | mean SOC Q10 [Mg ha^-1^] | se | Mean SOC Q90 [Mg ha^-1^] | se | n |  | ΔSOC [Pg] | se |  | ΔSOC [Pg] | se |
| arable | Alpine and Boreal arable | 8587.9 |  | 51.49 | 1.54 | 46.38 | 4.28 | 83.26 | 7.99 | 392 |  | -0.044 | 0.039 |  | 0.273 | 0.070 |
| arable | Atlantic and Central arable loamy and clayey | 71849.2 |  | 35.58 | 0.28 | 30.76 | 0.85 | 43.86 | 1.38 | 2583 |  | -0.346 | 0.064 |  | 0.594 | 0.101 |
| arable | Atlantic arable sandy | 2538.5 |  | 47.33 | 3.17 | 50.49 | 18.62 | 48.07 | 7.98 | 105 |  | 0.008 | 0.048 |  | 0.002 | 0.022 |
| arable | Central arable sandy | 3426.2 |  | 27.59 | 0.80 | 22.74 | 1.65 | 37.78 | 3.91 | 379 |  | -0.017 | 0.006 |  | 0.035 | 0.014 |
| arable | Mediterranean arable loamy and clayey | 22587.3 |  | 25.62 | 0.38 | 19.05 | 0.93 | 35.27 | 2.06 | 1003 |  | -0.148 | 0.023 |  | 0.218 | 0.047 |
| arable | Mediterranean arable sandy | 2.4 |  | 14.39 | 1.36 | 9.00 | 0.83 | 25.88 | 9.18 | 97 |  | 0.000 | 0.000 |  | 0.000 | 0.000 |
| grass | Cold climate semi-natural loamy and clayey | 17902.6 |  | 60.08 | 1.00 | 49.98 | 2.84 | 71.53 | 3.48 | 496 |  | -0.181 | 0.054 |  | 0.205 | 0.065 |
| grass | Cold climate semi-natural sandy | 912.1 |  | 65.27 | 4.35 | 71.39 | 17.05 | 80.24 | 16.21 | 69 |  | 0.006 | 0.016 |  | 0.014 | 0.015 |
| grass | Continental semi-natural loamy and clayey | 11529.6 |  | 53.88 | 0.88 | 36.74 | 2.35 | 66.59 | 2.46 | 637 |  | -0.198 | 0.029 |  | 0.146 | 0.030 |
| grass | Continental semi-natural sandy | 1012.3 |  | 46.20 | 2.28 | 47.65 | 7.69 | 49.76 | 6.43 | 152 |  | 0.001 | 0.008 |  | 0.004 | 0.007 |
| grass | Mediterranean arable loamy and clayey | 818.1 |  | 31.40 | 2.37 | 25.86 | 4.87 | 47.35 | 8.38 | 28 |  | -0.005 | 0.004 |  | 0.013 | 0.007 |
| grass | Southern semi-natural loamy and clayey | 2799.8 |  | 46.01 | 2.47 | 53.92 | 14.69 | 53.85 | 15.45 | 162 |  | 0.022 | 0.042 |  | 0.022 | 0.044 |
| grass | Southern semi-natural sandy | 0.2 |  | 22.70 | 2.50 | 17.18 | 2.01 | 26.44 | 2.40 | 37 |  | 0.000 | 0.000 |  | 0.000 | 0.000 |
| tree | Atlantic and Central arable loamy and clayey | 83.4 |  | 33.63 | 1.57 | 24.91 | 3.68 | 32.08 | 5.92 | 98 |  | -0.001 | 0.000 |  | 0.000 | 0.001 |
| tree | Mediterranean arable loamy and clayey | 5261.0 |  | 26.76 | 0.72 | 23.76 | 1.66 | 37.59 | 2.58 | 497 |  | -0.016 | 0.010 |  | 0.057 | 0.014 |
| tree | Mediterranean arable sandy | 0.2 |  | 18.02 | 1.43 | 18.03 | 5.40 | 26.01 | 4.59 | 45 |  | 0.000 | 0.000 |  | 0.000 | 0.000 |
|  |  |  |  |  |  |  |  |  |  | total: |  | -0.918 | 0.120 |  | 1.583 | 0.160 |

References

1. Feeney, C. J. *et al.* Benchmarking soil organic carbon (SOC) concentration provides more robust soil health assessment than the SOC/clay ratio at European scale. *Science of The Total Environment* **951**, 175642 (2024).

2. Fick, S. E. & Hijmans, R. J. WorldClim 2: new 1-km spatial resolution climate surfaces for global land areas. *International Journal of Climatology* **37**, 4302–4315 (2017).

3. Zomer, R. J., Xu, J. & Trabucco, A. Version 3 of the Global Aridity Index and Potential Evapotranspiration Database. *Sci Data* **9**, 409 (2022).

4. Beck, H. E. *et al.* High-resolution (1 km) Köppen-Geiger maps for 1901–2099 based on constrained CMIP6 projections. *Sci Data* **10**, 724 (2023).
